# Stimuli-Responsive Polymeric Nanoplatforms for Cancer Therapy

**DOI:** 10.3389/fbioe.2021.707319

**Published:** 2021-06-25

**Authors:** Di Chang, Yuanyuan Ma, Xiaoxuan Xu, Jinbing Xie, Shenghong Ju

**Affiliations:** Jiangsu Key Laboratory of Molecular and Functional Imaging, Department of Radiology, Zhongda Hospital, Medical School of Southeast University, Nanjing, China

**Keywords:** theranostic, nanomedicine, polymeric nanoparticle, stimuli-responsive, cancer therapy

## Abstract

Polymeric nanoparticles have been widely used as carriers of drugs and bioimaging agents due to their excellent biocompatibility, biodegradability, and structural versatility. The principal application of polymeric nanoparticles in medicine is for cancer therapy, with increased tumor accumulation, precision delivery of anticancer drugs to target sites, higher solubility of pharmaceutical properties and lower systemic toxicity. Recently, the stimuli-responsive polymeric nanoplatforms attracted more and more attention because they can change their physicochemical properties responding to the stimuli conditions, such as low pH, enzyme, redox agents, hypoxia, light, temperature, magnetic field, ultrasound, and so on. Moreover, the unique properties of stimuli-responsive polymeric nanocarriers in target tissues may significantly improve the bioactivity of delivered agents for cancer treatment. This review introduces stimuli-responsive polymeric nanoparticles and their applications in tumor theranostics with the loading of chemical drugs, nucleic drugs and imaging molecules. In addition, we discuss the strategy for designing multifunctional polymeric nanocarriers and provide the perspective for the clinical applications of these stimuli-responsive polymeric nanoplatforms.

## Introduction

Polymeric nanomaterials have gained much attention in medicine due to their unique advanced properties in cancer theranostics at the molecular level (Ren et al., 2016; Ekladious et al., 2019). Polymer molecules could be spontaneously self-assembled into nanomaterials under hydrophobic or electrostatic adsorption interactions. The polymeric nanocarriers with the loading of therapeutic drugs and imaging agents are promising to overcome the biological barriers for the theranostics of cancer (Li and Pu, 2020; Xu et al., 2021). Within these polymeric nanoplatforms, the designed smart polymeric nanocarriers responsive to the special stimuli conditions of tumor microenvironment have shown excellent effects in diagnosing and treating cancer (Montero de Espinosa et al., 2017; Wang F. et al., 2018). In particular, stimuli-responsive polymeric nanocarriers could enable the controlled release of drugs at the target sites. The distinct features of polymers to respond to the specific stimuli facilitate a high-throughput detection of molecular alterations as a result of the biological environment and allows regulation of pharmacokinetics of poorly soluble molecules, which becomes a novel trend for cancer therapy and should be engineered to realize different goals in the process of drug delivery (Li and Liu, 2014; Jiang et al., 2015). However, the multifunctional and stimuli-responsive nanocarriers also face several challenges, such as the need for better characterization, possible toxicity issues, limited absorption, and clinical transition of these nanocarrier-based delivery systems. Hence, a better understanding of the physiological environments-based stimuli of cancers and further improvement of the polymer-based nanocarrier systems are necessary for targeted therapeutic drug delivery applications. In this review, we focus on introducing stimuli-responsive polymer-based nanoplatforms and combined with imaging agents and drug/gene molecules for cancer treatment and diagnosis.

## Theranostic Nanomaterials

### Overview

Theranostic nanomaterials refer to the application of nanotechnology for both diagnosis and therapy in various diseases (Jain and Stylianopoulos, 2010; Xie et al., 2010; Bobo et al., 2016). As a rapidly evolving field combining nanotechnology, biomedical and pharmaceutical sciences, the progress of multifunctional nanocarriers has shown tremendous potential for enhancing therapeutics and diagnostics, especially for cancer treatment. Several nanomaterial-based drug delivery systems have already successfully improved the therapeutic profile of conventional drugs (Caster et al., 2017). Decreased toxicity and improved therapeutic effects are obtained by utilizing nanocarriers to increase selectivity by delivering chemicals or other agents toward a specific target (Table 1).

**TABLE 1:** Representative theranostic nanomaterials utilized in drug delivery system.

Type		Size (nm)	Pros	Cons
Liposomes		80–150	◆ Biocompatibility and biodegradability ◆ Ability to deliver both the hydrophilic and hydrophobic payloads ◆ Controlled pharmacokinetics and reduced toxicity ◆ Diverse surface modification	◆ Limited loading efficiency ◆ Limited stability *in vivo* ◆ Rapid clearance from the blood

Polymers	◆ Polymer conjugate complexes ◆ Polymer nanospheres ◆ Polymer micelles ◆ Dendrimers	1–20 10–200 20–200 3–50	◆ Tunable physiochemical properties ◆ Controllable size and composition ◆ Diverse surface modification ◆ High loading efficiency and sustained release ◆ Good circulation stability	◆ Limited storage stability ◆ Potential toxicity ◆ Limited capability for hydrophilic drugs ◆ Limited chemical synthesis

Iron oxide nanoparticles		varies	◆ Clinical used MRI contrast agent ◆ Magnetic hyperthermia and PAI ◆ Easy surface modification	◆ Limited stability under aqueous conditions

Quantum dots		2–10	◆ Unique optical properties ◆ Utilization for PDT	◆ Limited biodegradability and potential toxicity

Carbon nanotubes		0.8-exceed 100 nm (diameter) less than 100 nm-several cm (length)	◆ Strong optical absorbance and utilization for PTT, PAI ◆ Unique electrical property ◆ Easy surface modification	◆ Potential toxicity ◆ Limited biodegradability

Gold nanoparticles	◆ Gold nanosphere ◆ Gold nanorod ◆ Gold nanoshell ◆ Gold nanocage	5–150 20 nm-several μm 10–400 20–200	◆ Utilization for PTT, PAI, SERS ◆ Controllable size and structure and easy surface modification ◆ Optical quenching ability	◆ Limited stability under aqueous conditions

Upconversion nanoparticles		<100	◆ Unique optical property and utilization for luminescence imaging ◆ Utilization for PDT, PTT ◆ Easy surface modification and functionalization	◆ Potential toxicity ◆ Limited biodegradability

*MRI, magnetic resonance imaging; PTT, photothermal therapy; PAI, photoacoustic imaging; SERS, surface-enhanced Raman spectroscopy.*

Recently, the progress of stimuli-responsive nanomaterials has improved dramatically, especially in cancer treatment. The stimuli can be divided into internal and external stimuli. The internal stimuli generally include pH, redox potential, enzymes, and hypoxia (Fleige et al., 2012), while the external stimuli include light, magnetic field, ultrasound, temperature, radiation, and others (Karimi et al., 2016). The unique properties of nanomaterials enable them to respond to the stimuli, realizing different goals in diagnosing and drug delivery systems. Stimuli-responsive nanomaterials will become a new trend, and more novel nanoparticles should be engineered for treating cancers.

### Theranostic Platforms

The most commonly used theranostic nanoplatforms in basic research and clinical practice are liposomes (Al-Jamal and Kostarelos, 2011; Wen et al., 2012), inorganic nanomaterials (Cabral et al., 2014; McHugh et al., 2018), and polymeric nanoparticles (Wang Z. et al., 2014; Kamaly et al., 2016), which are extensively employed theranostic nanocarriers in cancer treatment.

**FIGURE 1:**
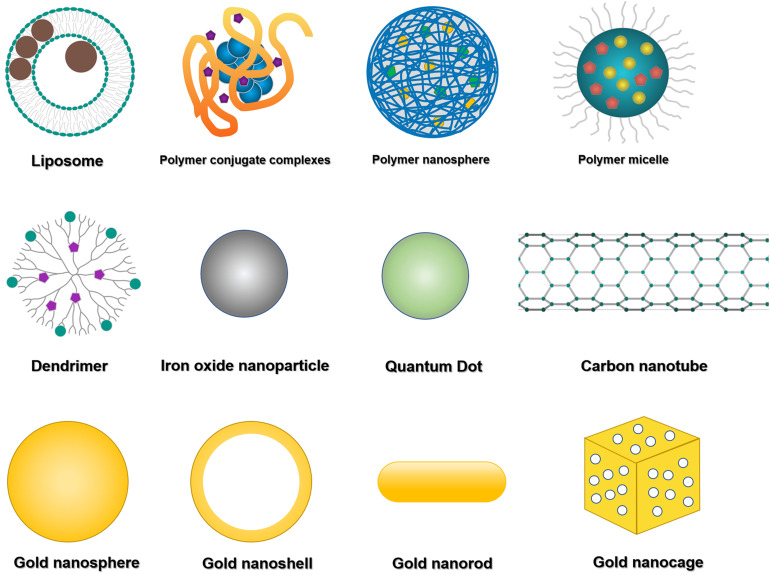
Representative theranostic nanomaterials. The most commonly used theranostic platforms in basic research and clinical practice are liposomes, polymeric nanoparticles (mainly including polymer conjugate complexes, polymeric nanospheres, polymeric micelles, and dendritic polymers), and inorganic nanomaterials (mainly including iron oxide nanoparticles, quantum dots, carbon nanotubes, and various kinds of gold nanoparticles).

Liposomes (Figure 1) are bilayered phospholipid vesicles that can self-close to form spheres (Xing et al., 2016; Carita et al., 2018). Due to their size, biocompatibility, biodegradability, low immunogenicity and toxicity, and the encapsulating capacity for hydrophilic and hydrophobic agents, liposomes have been well applied in preclinical studies as drug and imaging agent carriers. The liposomal formulation is the first nanomedicine approved by the United States Food and Drug Administration (US FDA) for clinical application. The best application of liposomal formulation in the clinic is liposomal doxorubicin (DOX), which encapsulates DOX inside of the aqueous core and shields by polyethylene glycol (PEG) to overcome opsonization, prolong systemic drug circulation, improve therapeutic efficacy, and have been used for the treatment of Kaposi’s sarcoma, ovarian cancer and multiple myeloma (Xing et al., 2016). Grange et al. evaluated the therapeutic efficiency of DOX-loaded liposomes in Kaposi’s sarcoma model, and tracked the liposome tissue distribution as well as monitored drug release by *in vivo* magnetic resonance imaging (MRI) (Grange et al., 2010). Wen et al. evaluated the brain targeting theranostic liposomes loaded with quantum dots and apomorphine (Wen et al., 2012). They studied the distribution of theranostic liposomes by visualizing the fluorescence derived from quantum dots and found a significant increase in the accumulation of theranostic liposomes in the brain compared with free quantum dots.

Numerous inorganic nanomaterials have been investigated for biomedical applications because of their physical functions, such as magnetic properties of iron oxide nanoparticles, light emission of quantum dots, and optical and thermal properties of gold nanoparticles (Vigderman and Zubarev, 2013; Sharma et al., 2015; Her et al., 2017; He et al., 2021). These nanomaterials can be prepared in ultra-small sizes, susceptible to renal excretion (Prasad, 2012; Cabral et al., 2014). Currently, iron oxide nanoparticles (IONPs) (Figure 1) are nanocrystals made from magnetite or hematite and have been widely utilized as a T2WI contrast agent due to their T2 substantial relaxation rate effect for *in vivo* tumor MRI (Xie et al., 2010). Quantum dots (QDs) (Figure 1) are semiconductor nanocrystals with available diameters ranging from 2 to 10 nm. They are typically composed of Groups II-VI such as CdSe and CdTe, III–V such as InP and InAs (Prabhu and Patravale, 2012). QDs exhibit optical properties with less photobleaching, longer photoluminescence lifetime, and brighter fluorescence than other fluorophores. Gold nanoparticles (AuNPs) (Figure 1) are helpful for cancer diagnosis and photothermal therapy because of their optical properties (Sharma et al., 2015). They can be synthesized in different sizes and shapes such as spherical, rod-like, cage, or even irregular shapes (Vigderman and Zubarev, 2013). In addition, AuNPs can also be utilized as computed tomography (CT) contrast agents. Carbon nanotubes (CNTs) (Figure 1) are cylinder nanomaterials consisting of one or more graphene layers. CNTs can be single-walled (SWCNTs) with a diameter typically 0.8 to 2 nm and length ranging from less than 100 nm to several centimeters. Another form of CNTs is multi-walled (MWCNTs) with a 5 to 20 nm diameter and can exceed 100 nm (De Volder et al., 2013). CNTs have thermal, mechanical and electrical properties related to their structure, stability, ease for modification and morphology (Kumar et al., 2017), thus having a potential application in Raman and photoacoustic imaging and drug delivery (Wong et al., 2013).

Polymeric nanomaterials have been widely used as carriers of drugs and bioimaging agents because of their excellent biocompatibility, biodegradability and structural versatility (Luk and Zhang, 2014; Wang Z. et al., 2014; Butowska et al., 2021). Polymers could simultaneously self-assemble into polymeric nanoparticles with encapsulating therapeutic drugs or imaging agents, thus enabling multiple functions in one nanosystem to meet the theranostic requirements. Polymeric nanomaterials such as PEG, poly(D, L-glycolic acid), and poly(D, L-lactic acid) have already been approved for clinical application (Luk and Zhang, 2014). With different nanomaterials, polymers possess different capabilities, including enhanced drug efficacy than free drugs by improved drug encapsulation and delivery, prolonged circulating half-life and triggered drug release, and so on (Wong and Choi, 2015; Luque-Michel et al., 2017). For example, by coating with PEG, they can circulate for a prolonged circulating time in the blood, avoid quick recognition and elimination by the immune system, then gradually release drugs in tumors and simultaneously facilitate tumor imaging. Polymers can still accumulate in the targeted areas of diseased tissues by either passive targeting via enhanced permeability and retention (EPR) effect or active targeting via cell surface ligands/receptors (Luk and Zhang, 2014).

Polymeric nanomaterials can also combine their unique properties with other modalities of theranostic agents, such as combined with inorganic nanomaterials to form polymer-based hybrid nanomaterials. For example, IONPs surface-modified with targeting ligands or conjugated with polymers can be monitored in real-time through MRI, improving active accumulation at the lesion sites (Caldorera-Moore et al., 2011; Wang Z. et al., 2014; Sharma et al., 2017). These polymer-based nanoparticles possess a powerful theranostic vehicle in both preclinical and clinical use. So far, the distinctive properties of polymeric nanomaterials have led to their extensive research and application in cancer therapy.

More importantly, the stimuli-responsive polymeric nanoparticles attract much attention, as they can alter their physicochemical properties responding to external stimuli, such as temperature, light, enzyme, and pH changes. After stimulation, the volume, interior network permeability, or hydrophilicity-hydrophobicity of the nanoparticles are possibly changed, leading to imaging agents or drugs/genes release to generate signals for imaging or affecting cell functions. For example, pH-responsive polymeric nanoparticles could be stimulated at pH 5.7–7.0 in the solid tumor microenvironment, stable at pH 7.4 in the blood. Bae et al. synthesized pH-responsive polymeric nanoparticles using poly(L-histidine)-PEG block copolymer for cancer treatment (Prasad, 2012; Cabral et al., 2014). The hydrophobic poly histidine (PHis) was the pH-responsive moiety, which can become hydrophilic by protonation at low pH to induce drug release. These unique characteristics make polymeric nanoparticles ideal nanocarriers for tumor-targeted drug delivery.

### Passive and Active Targeting of Nanomaterials

In past decades, many responsive theranostic nanomaterials have been developed to control the release and the rate of loading drugs in cancer treatment (Choi et al., 2019; Zhang et al., 2020). Theranostic nanomaterials carry therapeutic agents to the target tissues and release them to kill the diseased cells. The degree of drug delivery efficacy is highly dependent on the structures and properties of the nanomaterials. The effective localization and release rate of nanomaterials to tumor sites are mainly achieved through passive or active targeting of controlled chemicals or drugs to diseased tissues (Bertrand et al., 2014; Wang et al., 2016b, c).

#### Passive Targeting

Passive targeting in cancer refers to the preferential accumulation of nanoparticles to the tumor tissues. Due to the leaky tumor vasculature and impaired lymphatic drainage, and the unique microenvironment surrounding the cancer cells, theranostic nanomaterials can accumulate and be retained in tumor tissues longer than in normal tissues, which is also called EPR effect (Overchuk and Zheng, 2018; He et al., 2019; Maeda, 2021). Passive targeting is directly associated with the nanoparticles’ inherent properties, including size, shape, charge, flexibility, *etc*. Nanotechnology has accelerated the development of polymeric drugs for cancer therapy because polymeric nanomaterials can alter the physicochemical features such as size, shape and charge potential to enhance the EPR effect directly or indirectly (Maeda et al., 2009; Huang et al., 2021). The typical size of nanosystems ranges from 5 to 200 nm to avoid kidney filtration and extravasate the leaky vasculature in tumor (Dai et al., 2017). The size of nanomaterials can affect the penetration rates in solid tumors and influence the biodistribution and tumor accumulation behavior *in vivo* (Schadlich et al., 2011). In addition, the shape can also affect the properties of nanomaterials. Nanomaterials with different shape characters such as spherical, cubic, star-like have been designed in the drug delivery system, which can influence the cellular uptake and efficacy of loading drugs. The surface charge is another crucial parameter for the design and synthesis of nanomaterials. Positively charged nanomaterials show a higher affinity to cells and enhance cellular uptake due to the electrostatic interaction between negatively charged cells surface. However, positively charged nanomaterials have relatively high systemic toxicity and are more vulnerable to be cleared by the mononuclear phagocyte system, limiting their applications. In contrast, the neutral and negatively charged nanomaterials can avoid the non-specific interactions with proteins in the blood and have an extended circulation period.

#### Active Targeting

Active targeting is developed to enhance the accumulation of nanomaterials at the target sites in tumor tissues as a complementary strategy of passive targeting (Rosenblum et al., 2018; Ganguly et al., 2021; Wang et al., 2021). The theranostic nanomaterials modified with affinity ligands (antibodies, proteins, peptides, nucleic acids, aptamers or small molecules) could be selectively recognized by the receptors expressed on the target cells, or tissues could be delivered to the subcellular locations through an endocytosis pathway (Tanner et al., 2011; Choi et al., 2019; Mi et al., 2020). The targeting specificity and the delivering capacity are known as the two principal features in evaluating the targeting efficiency of nanomaterials (Bertrand et al., 2014). Recently, Shmidt et al. found that for nanomaterials with relatively large sizes (diameter ≥ 50 nm), active targeting does not significantly increase the tumor localization than non-targeted nanomaterials. However, the incorporation of targeting ligands on the surface of nanomaterials increases their cellular internalization by the target cells in the tumor, which is a prominent role of active targeting nanomaterials (Schmidt and Wittrup, 2009). Thus, active targeting has been utilized to enhance the delivery of high molecular weight molecules (macromolecules, e.g., proteins, RNA, DNA, *etc*.) to their target cells. For example, when the nanoparticles are functionalized with these targeting ligands, they can recognize the receptors on the target cells and bind via receptor-ligand interactions, whereby they are internalized through ligands-mediated endocytosis (Sahay et al., 2010). After cellular internalization, these nanoparticles trigger anticancer drugs inside the cancer cells based on biological stimuli, leading to cell death (Rosenblum et al., 2018).

## Polymeric Nanomaterials

Polymeric nanoparticles are organic-based nanomaterials and have been explored widely as theranostic agents due to the plethora of benefits and significant efficacy in cancer treatment (Luk et al., 2012; Senapati et al., 2018; Zielinska et al., 2020; Mitchell et al., 2021). Various subtypes of polymeric nanomaterials have been developed to aid in drug delivery to cancerous sites, mainly including polymer conjugate complexes, polymeric micelles, polymeric nanospheres, and dendritic polymers (Luk and Zhang, 2014).

### Polymer Conjugate Complexes

Conjugation of polymeric macromolecules with drugs and functional imaging agents to form polymer conjugate complexes is a new paradigm for delivering drugs and imaging agents, improving the solubility of hydrophobic molecules, prolonging their circulation time *in vivo*, and enhancing their specific accumulation in tissues (Figure 1). N-(2-Hydroxypropyl) methacrylamide (HPMA), poly (lactic-co-glycolic acid) (PLGA), poly (lactic acid) (PLA) and poly glycolic acid (PGA) are the commonly used polymers to synthesize nanoparticles because of their stability and biocompatibility (Prabhu and Patravale, 2012). For example, Li et al. conjugated PGA with gadolinium (Gd) and paclitaxel and imaged tumor necrosis after administration using MRI (Jackson et al., 2007); Lu et al. monitored the therapeutic efficacy of photodynamic therapy (PDT) on xenograft tumors by administration of PGA-photo-sensitizer/Gd conjugates with contrast-enhanced MRI (Vaidya et al., 2006).

### Polymeric Micelles

Polymeric micelles are an essential subtype of polymeric nanoparticles, self-assembled structures with a hydrophobic core and hydrophilic exterior with an approximate size range of 20–200 nm (Zhou et al., 2018; Figure 1). They have been widely used in theranostic systems for cancer therapy owing to unique biocompatibility, high solubility and longer circulation time *in vivo* (when crosslinked). For instance, Wan et al. have developed a synergistic method with both photothermal therapy and chemotherapy capabilities using cyanine dye and DOX-loaded polymeric micelles in mice with lung cancer, which achieved better synergistic therapeutic efficacy compared to a single therapy (Wan et al., 2014). The micelles were triggered successfully by photo-irradiation, which caused photothermal damage to tumor cells and led to cytotoxic damage induced by DOX simultaneously. General-PM^TM^ is the best example of a clinical polymeric micellar nanoparticle for cancer therapy, which encapsulated paclitaxel in a polymeric micelle formed by monomethoxy poly(ethylene glycol)-block-poly(D, L-lactide) (Oerlemans et al., 2010; Martinelli et al., 2019). However, due to the dynamic behavior of micelles and the existence of critical micellar concentration, micelles often face challenges such as lower *in vivo* stability and poor drug loading capacity when applied in theranostic systems, which call for improved nanotechnology in optimizing the physicochemical features of micelles.

### Polymeric Nanospheres

Polymeric nanospheres possess a predominantly hydrophobic feature to achieve an optimal nanosphere loading (Shim et al., 2004; Guo et al., 2015; Figure 1). Polymeric nanospheres could be spontaneously assembled by themselves in aqueous media with hydrophobic blocks in the core and hydrophilic blocks outside. As a result, hydrophobic drug or imaging agents could be encapsulated in the core, while hydrophilic small molecular therapeutics and macromolecules, such as proteins and nucleic acids, could be loaded corona. As different hydrophobic and hydrophilic blocks with various charges, lengths and structures, have been utilized to form polymeric nanospheres for drug and imaging agent delivery, the sizes, shapes, and stabilities of polymeric nanospheres were different. Most have relatively narrow size distributions with diameters ranging from 10 to 200 nm. Boltnarova et al. have prepared polymer nanospheres based on PLGA with low molar weight for macrophage-targeted drug delivery using both nanoprecipitation and emulsification solvent evaporation methods, which serves as a compelling, biodegradable and biocompatible drug delivery platform for macrophages (Boltnarova et al., 2021).

### Dendritic Polymers

Dendritic polymers are highly branched polymers with controllable structures and many terminal functional groups (Xie et al., 2010; Rizzo et al., 2013; Ma et al., 2016; Figure 1). With three-dimensional architectures, various application-related properties of dendritic polymers, such as self-assembly, biodegradability, biocompatibility, and stimuli-responsiveness ability, have been adjusted and controlled through synthetic procedures. To date, progress has been made for dendritic polymers in solving fundamental and technical problems toward their theranostic applications. Ma et al. classified at least six subclasses, including dendrimers, hyperbranched polymers (HBPs), multi-arm star polymers, dendronized or dendrigraft polymers, hypergraphs or hypergrafted polymers, and dendritic-linear block polymers (Ma et al., 2016). Among them, dendrimers and hyperbranched polymers and the two major subclasses of dendritic polymers. Dendrimers are an important class of dendritic polymers known for their well-defined spherical-shaped structures, high functionality, and versatile drug delivery capabilities (Madaan et al., 2014). Dendrimers have potential abilities in entrapping and conjugating various hydrophilic/hydrophobic entities by host-guest interactions, and the high surface group functionality, tunable size and low polydispersity have made them ideal candidates for theranostic applications. For instance, Yousef et al. have successfully applied galactosamine targeted G4 polyamidoamine dendrimer to fulfill the efficient delivery of anticancer curcumin derivative for hepatocellular cellular carcinoma treatment (Yousef et al., 2018). HBP is another class of dendritic polymers with ill-defined structures and can merge multiple functionalities into a single entity (Ma et al., 2016). HBPs have unique advantages of facile one-pot fabrication (Zheng et al., 2015). Compared with other polymeric variants, the high end-group functionality and structural versatility of HBPs allow the attachment of a higher density of targeting ligands via non-covalent or covalent interactions, which can trigger stimuli-responsive drug release on the target site.

### Biophysicochemical Features of Polymeric Nanomaterials

Polymeric nanomaterials can alter their physicochemical features such as size, shape and charge potential to enhance the EPR effect directly or indirectly (Maeda et al., 2009). For example, Schädlich et al. investigated the influence of size on the biodegradable polymeric nanoparticles (Schadlich et al., 2011). The nanoparticles were synthesized by polyethylene glycol-polyesters poly(lactide) block polymers (PEG-PLA) loading near-infrared (NIR)-dye which could be used to evaluate the distribution *in vivo*. Three PEG_2_-PLA_20_ or PEG_2_PLA_40_ (numbers in kDa) nanoparticle formulations with different and defined sizes were tested at two different xenograft tumor types, the HT29 (colorectal carcinoma) and the A2780 (ovarian carcinoma) cell lines in the research. The results showed that nanoparticles with 111 nm and 141 nm in diameter could efficiently accumulate in the tumor tissue, while the slightly larger nanoparticle whose diameter was 166 nm tended to be eliminated by the liver. Rampersaud et al. investigated the influence of shape on the drug release and anticancer efficacy of IONPs (Rampersaud et al., 2016). They used IONPs capped by dextran, a neutral and hydrophilic polymer, with a cage shape or a solid spherical shape, respectively loading riluzole, and found that the anticancer efficacy increased 3-fold in LM7 cells with the cage-shaped IONPs. The porous nature of dextran allows drugs to be released at a controlled rate, and the difference for anticancer efficacy was mainly based on the surface charge caused by different shapes of nanomaterials. The charge of riluzole-incorporated cage-shaped IONPs was more damaging than the spherical ones, leading to a longer time for riluzole to block membrane ion channels and kill more cancer cells apoptosis.

Additionally, Ramos et al. investigated the influence of cationic surface charge of the polymeric nanoparticles (Ramos et al., 2014). Polyethylene imine (PEI), a typical example of cationic polymer nanoparticles, showed increased membrane permeability with repeating units of amine groups. The positively charged nanomaterials can interact with the negatively charged gene, which could be entrapped or conjugated in the polymer nanosystem. Nevertheless, positively charged nanomaterials have some limitations, such as systemic toxicity. In contrast, neutrally and negatively charged nanomaterials have advantages in avoiding non-specific interactions and prolonging circulation time. Therefore, multiple cancer microenvironmental stimuli-responsive nanomaterials have been developed, which could alter their physicochemical features, such as reverse the surface charge and release the loading agents at the target sites to enhance drug/gene delivery (Han et al., 2012; Yuan et al., 2012; Amin et al., 2015).

## Stimuli-Responsive Polymeric Nanoparticles for Cancer Therapy

### Cancer Therapy and Stimuli-Responsive Microenvironment

Cancer is one of the most important public health problems and the leading cause of death worldwide. Data from GLOBOCAN in the year 2020, about 19.3 million new cancer cases and 10.0 million cancer deaths lead to a considerable burden on society all over the world (Sung et al., 2021). The therapeutic methods employed globally for cancer treatment are surgery, chemotherapy (CHT), radiation therapy (RT) and immunotherapy (Luque-Michel et al., 2017). Surgery is the primary treatment modality for most solid tumors (Nguyen and Tsien, 2013). However, not all tumors can be removed via surgery due to their progression and stages, and surgical margins cannot be eradicated because of the poor differentiation from normal tissues. CHT and RT have shown their success in suppressing the proliferation and increasing the survival rate of patients, but the efficacy of CHT and RT is far from satisfactory due to the high toxicity and the damage of healthy tissues. Immunotherapy only works in a subset of cancers, and the percentage of patients who respond is low (Nguyen and Tsien, 2013; Yang, 2015). Thus, some breakthroughs should be made in the field of cancer treatment.

**FIGURE 2:**
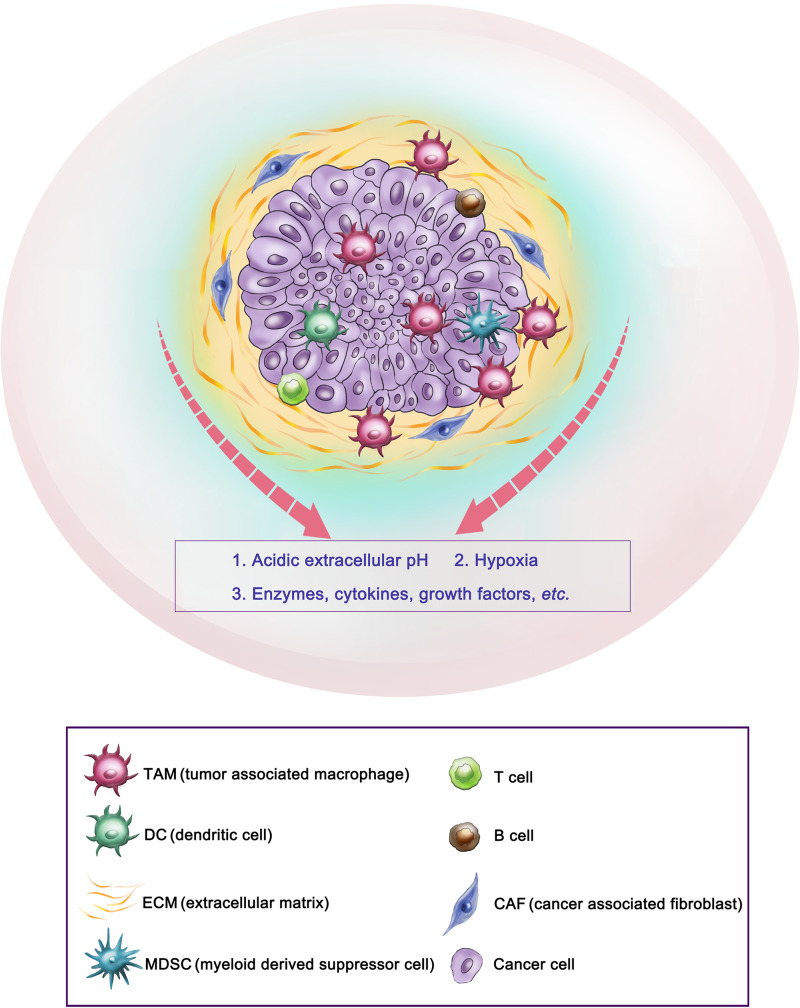
Representative tumor microenvironment of a solid tumor. This scheme shows the representative tumor microenvironment of a solid tumor (pancreatic ductal adenocarcinoma). The tumor microenvironment in solid tumors commonly consists of tumor-associated macrophages (TAM), cancer-associated fibroblasts (CAF), extracellular matrix (ECM), *etc.*, and abnormal physiological environments such as acidic extracellular pH and hypoxia, as well as overexpressed enzymes, cytokines, *etc.*

The tumor microenvironment (TME) is widely known as a main contributor to the development and progression of many cancers. TME in solid tumors mainly consists of immune cells, such as tumor-associated macrophages, dendritic cells, T and B lymphocytes; stromal cells such as cancer-associated fibroblasts and mesenchymal stromal cells; extracellular matrix and other secreted molecules, such as enzymes, cytokines, growth factors, *etc*. In addition, abnormal physiological environments, such as acidic extracellular pH and hypoxia, also play key roles in cancer progression, metastasis and drug resistance (Spill et al., 2016; Wang Y.A. et al., 2018; Bejarano et al., 2021; Figure 2). For example, the extracellular pH in tumor tissues is more acidic (5.7–6.9) than in the blood pH (7.4) at 37°C (Alfarouk et al., 2011). Compared to normal tissues, physiochemical properties in solid tumors are largely different, such as temperature is higher, oxygen partial pressure is reduced (hypoxia), and many enzymes and cytokines are overexpressed in the TME (Li et al., 2014; Chen et al., 2016; Hao et al., 2017).

### Theranostic Polymeric Nanomaterials for Cancer

The field of drug delivery systems becomes popular in recent years by using synthetic polymers for drug development in cancer therapy. These polymer-based new drug entities are called “polymer therapeutics,” and theranostic polymeric nanomaterials have already been utilized in numerous cancers for drug delivery (Duncan, 2003; Duncan et al., 2005; Vicent and Duncan, 2006). In general, polymer nanomedicines are designed to improve drug performance by utilizing pathophysiological characteristics of solid tumors, of which conventional low molecular weight drugs are incapable. Improved tumor-selective targeting of polymer nanomedicines and macromolecular drugs is shown due to the prolonged circulation time of these nanoparticles, leading to improved therapeutic efficacy and fewer side effects (Duncan, 2006; Greco and Vicent, 2009). Polymer-based theranostic nanomaterials can load therapeutic agents to targeted tissues or cells and regulate the release of drugs at a customized dose and time, increasing the therapeutic efficiency and reducing the side effect.

In particular, stimuli-responsive features of the polymeric nanoparticles would make an unprecedented control over the delivery and release of therapeutics at the disease site (Ke et al., 2019). Hence, developing stimuli-responsive polymeric nanoparticles that can specifically respond to TME offers promising strategies for combating cancer (Jiang et al., 2020; Qi et al., 2021). Recently, the progress of stimuli-responsive nanomaterials has improved dramatically in cancer treatment (Fleige et al., 2012; Yu J. et al., 2014; Karimi et al., 2016; Alsehli, 2020; Pham et al., 2020; Xie et al., 2020). The stimuli can be divided into internal and external stimuli. The internal stimuli generally include pH, redox potential, enzymes, hypoxia, *etc*. In contrast, the external stimuli include light, magnetic field, ultrasound, temperature, radiation, *etc.* (Karimi et al., 2016). After stimulation, the physicochemical features of the nanoparticles, such as the interior network permeability or hydrophilicity-hydrophobicity, are changed, which lead to imaging agent or drug/gene release to target sites. Thus, the following part will introduce different types of stimuli and the applications of their corresponding stimuli-responsive polymeric nanomaterials that expand the biomedical applications of theranostic nanomaterials.

### Internal Stimuli

#### pH-Responsive Polymers

**FIGURE 3:**
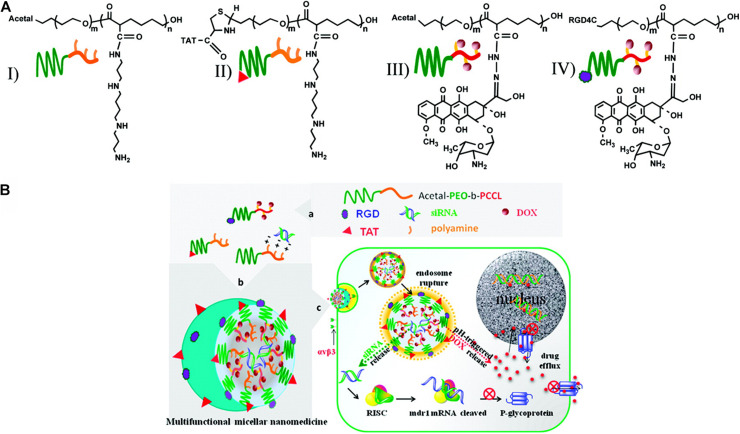
Schematic representation of multifunctional micellar nanocarriers triggered by acidic pH. **(A)** Schematic illustration of acetal- and TAT-PEO-bP(CL-g-SP) (I and II) and acetal- and RGD4C-PEO-bP(CL-Hyd- DOX) (III and IV). **(B)** Rational design of a multifunctional micellar nanomedicine for cancer-targeted co-delivery of MDR-1 siRNA and DOX to overcome multidrug resistance. DOX release from NON-micelles triggered by acidic pH. Reprinted from Xiao-Bing Xiong and Afsaneh Lavasanifar. Traceable Multifunctional Micellar Nanocarriers for Cancer-Targeted Co-delivery of MDR-1 siRNA and Doxorubicin. ACS Nano. 2011;5(6):5202–13. With the permission of ACS publications/from reference [105]. MDR, multidrug resistance; PEO, poly(ethylene oxide); RGD, Arg-GLT-Asp (the integrin αvβ3-specific ligand); DOX, doxorubicin; TAT, trans-activating transcriptional activator.

Appreciable pH variation is one of the most commonly used factors for the design of stimuli-responsive nanomaterials. Because of the abnormally fast metabolism and proliferation, a great amount of lactic acid and some end-products were produced by tumor cells, which may induce toxic effects to the adjacent tissue and an acidic pH ranging from 5.7–6.9 (Liu J. et al., 2014). Thus, many responsive polymer nanoparticles are designed to deliver drugs or genes and control release at the target sites in cancer treatment (Du et al., 2015; Kanamala et al., 2016; Li et al., 2016). For example, Chang et al. developed a polymer micelle consisting of poly[(D,L-lactide)-co-glycolide]-PEG-poly[(D,L-lactide) coglycolide] copolymer capped with N-Boc-histidine (Chang et al., 2010). Modification with N-Bochistidine enhanced the biodegradability and biocompatibility of the micelles, and DOX was loaded into micelles as an anticancer drug. Compared to pH 7.4 of normal tissues, the acidic pH microenvironment in breast cancer triggered significantly higher DOX release at pH 6.2. The pH-sensitive polymer nanoparticles released anticancer drugs with lower systemic toxicity compared with free drugs. The drugs should be released rapidly from the polymeric nanosystems under an acidic pH microenvironment in the tumor cells to improve the pharmacological effects of drug-loaded polymers and reduce multidrug resistance. Polymeric nanosystems that can maximize intracellular drug delivery and minimize drug release in the extracellular space are preferred. Hu et al. used PEG-cis-aconityl-chitosan-stearic acid polymeric micelles for pH-trigged DOX release, which reduced cytotoxicity due to the high internalization of the micelles into the tumor cells (Hu et al., 2012). In another study, Yu et al. designed polymeric micelles based on PbAE, altering their size and surface charge at tumor sites (Yu Y. et al., 2014). The micelles were synthesized by poly(ethylene glycol)-poly(lactide)-poly(β-amino ester) (MPEG-PLA-PAE) copolymers. In the circulation system, the micelles remained a larger size and were composed of a hydrophobic PLA/PAE core and hydrophilic PEG shell. When the micelles were exposed to the acidic environment, the tertiary amine group in the PAE underwent protonation and switched from hydrophobic to hydrophilic, leading to a shrinking size to 20–30 nm the release of the loading drugs. This important change caused a lower diffusional hindrance in the interstitial matrix and an improved cellular uptake of the tumor tissues. Zhao et al. reported mixed micelles consisting of poly[(D, L-lactide)-co-glycolide]-PEG-folate (PLGA-PEGFOL) and poly (b-amino ester)-poly(ethylene glycol)-folate (PAE-PEG-FOL) for endosomal pH-triggered DOX release (Zhao et al., 2010). These polymer micelles also showed improved cytotoxicity, which is attributed to the specific binding of the ligands of micelles to the cell membrane, and the micelles are internalized by endocytosis. In another study, Xiong et al. reported a kind of pH-responsive polymeric micelles that could deliver siRNA and chemotherapeutic agent DOX in one system simultaneously (Xiong and Lavasanifar, 2011; Figure 3). A micellar system was constructed from degradable poly(ethylene oxide)-block-poly(ε-caprolactone) (PEO-b-PCL) block copolymers with functional groups on both blocks. The functional group on the PCL block was used to incorporate short polyamines for complexation with siRNA or to chemically conjugate DOX via a pH-sensitive hydrazone linkage. The DOX could be released in cancer cells via a pH-sensitive hydrazone linkage in the acid environment. With the combination of siRNA delivery, the P-glycoprotein expression could be inhibited, leading to the inhibition of P-GP-mediated DOX resistance in MDA-MB-435 tumor models. Additionally, this kind of nanocarriers could incorporate fluorescent probes in the micellar core to track the siRNA so that the theranostic goals could be achieved (Xiong and Lavasanifar, 2011). Moreover, pH alterations can modulate the imaging state of nanomaterials and trigger anticancer therapy. Ling et al. developed a new class of nanomaterials composed of self-assembled IONPs and pH-responsive ligands (Ling et al., 2014). This multifunctional system consists of a pH-sensitive polymer, which could target the cancerous tissues through surface-charge switching induced by the acidic extracellular microenvironment and extremely small IONPs that can disassemble into the cancer cells, causing a significant MR contrast effect as well as a photosensitizer with fluorescence and photodynamic therapeutic ability. Because of a lower pH in the subcellular compartments, the photosensitizers were exposed and generated the singlet oxygen to enable the photodynamic therapy to kill cancer cells selectively. These pH-responsive nanoparticles showed superior therapeutic efficacy in highly heterogeneous drug-resistant tumors (Ling et al., 2014). However, the bioavailability of these nanomaterials still requires to be improved, and the response rate to the pH stimulus must be tuned for proper applications (Liu J. et al., 2014).

#### Redox-Responsive Polymers

**FIGURE 4:**
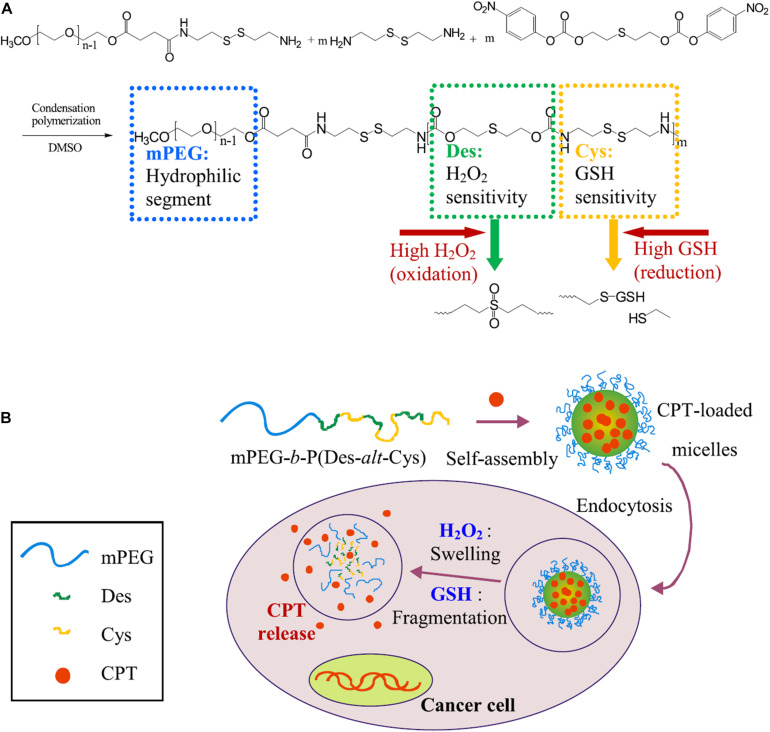
Schematic illustration of dual redox-responsive micelles. **(A)** Chemical structure of mPEG-b-P(Des-alt-Cys) copolymer and **(B)** dual redox-responsive micelles and CPT release triggered by ROS and GSH. The dual redox-responsive micelles enter into cancer cells and exhibited high levels of ROS and GSH, then the structures of micelles are deformed, and the encapsulated CPT could be liberated from micelles, leading to selectively location-controlled drug release. Reprinted from Yi-Ting Chiang, Yu-Wei Yen, and Chun-Liang Lo. Reactive oxygen species and glutathione dual redox-responsive micelles for selective cytotoxicity of cancer. Biomaterials. 2015;61:150–61. With permission from reference (Chiang et al., 2015). PEG, poly(ethylene glycol); Des, diethyl sulfide; Cys, cystamine; CPT, camptothecin; ROS, reactive oxygen species; GSH, glutathione.

Redox potential is another property that can control the release of loading drugs in polymeric nanoparticle delivery systems (Zhang et al., 2017). Similar to the pH, a gradient of redox potential exists between healthy and cancerous tissues and intracellular and extracellular compartments, which leads to the development of redox-responsive nanomaterials (Han et al., 2017). For example, the level of glutathione tripeptide(γ-glutamyl-cysteinyl-glycine) (GSH) in tumor tissues is at least four times higher than that in normal tissues (Karimi et al., 2016; Thambi et al., 2016). In addition, the intracellular concentration (2–10 mM) of GSH is about 100–1000 times higher than that in extracellular compartments (2–10 μM) (Han et al., 2017). Therefore, many redox-responsive nanomaterials have been developed with the ability to trigger the release of therapeutic agents. Wang et al. developed an amphiphilic polyanhydride copolymer containing disulfide bonds between the hydrophilic and hydrophobic segments (Wang J. et al., 2014). The copolymer can self-assemble into stable micelles with well-defined core-shell structure, and GSH triggered the disassembly behaviors of the micelles. These micelles showed excellent efficiency in inhibiting the growth of cancer cells in 4T1 tumor-bearing BALB/c mice due to the rapidly intracellular delivery of therapeutic agents. Quantitative analysis revealed that the redox-responsive micelles had enhanced therapeutic effects in solid tumors compared with the redox-insensitive micelles. In addition, redox-sensitive prodrug polymeric nanoparticles exhibit a unique advantage in overcoming multidrug resistance (MDR) and improving the overall therapeutic efficiency of anticancer drugs in cancer treatment. Liu et al. developed a redox-responsive DOX prodrug by conjugating DOX to DEX-PEI polymers via disulfide linkers (Liu et al., 2013). The prodrug self-assembled into polymeric micelles with an average size of 100–140 nm and exhibited a rapid drug release rate under the intracellular reduction environment (10 mM DTT). In the absence of DTT, a minimal amount of DOX was released within 48 h; however, around 50% of DOX was released within 4 h in 10 mM DTT. Additionally, the redox-responsive prodrug micelles enhanced the cellular accumulation of the DOX and achieved endosomal escape in human breast cancer multidrug-resistant cells (MCF-7/ADR) compared to free DOX. In another study, Han et al. developed self-assembled redox-responsive polymeric nanoparticles based on hyaluronic acid (HA)-polycaprolactone (PCL) block copolymer as drug carriers for cancer therapy (Han et al., 2015). The HA shell was crosslinked via a disulfide linkage. The anticancer drug DOX was efficiently encapsulated into the nanoparticles with a high drug loading rate. The DOX-loaded HA nanoparticles significantly retarded the drug release under physiological conditions (pH 7.4). The drug release rate showed a marked increase in the existence of GSH bonds in the cytoplasm. Improved antitumor efficacy was investigated using such tumor-targeted crosslinked polymeric nanoparticles than non-cross-linked nanoparticles and free chemotherapeutic drugs. In addition, Chiang et al. generated the dual redox-responsive micelles for selective cytotoxicity of cancer (Chiang et al., 2015; Figure 4). This kind of micelles could release the anticancer drug camptothecin in the cancer cells after the explosion of reactive oxygen species (ROS) and GSH. ROS is another essential factor in controlling the balance of redox in cancer cells, and the concentration in tumor tissues is about 100 times higher than that in normal cells because of the oncogene stimulation, mitochondrial malfunction and chronic inflammation. The ROS-responsive diethyl sulfide of the micelles could cause a swollen effect, and the GSH-responsive disulfide-containing cystamine further promoted the process of copolymer fragmentation, which led to the release of drugs in cancer cells. Redox-responsive polymeric nanoparticles can also be used for effective gene delivery. Jia et al. synthesized the chitosan oligosaccharide-based disulfide-containing polyethyleneimine derivative PEG-ss-COS-ss-PEI as a non-viral gene delivery carrier (Jia et al., 2013). The achieved PEG-ss-COS-ss-PEI copolymers could effectively condense DNA into small particles with an average diameter smaller than 120 nm. In the existence of 10 mM GSH, polyplexes of PEG-ss-COS-ss-PEI were rapidly unpacked, as revealed by a significant increase of particle sizes to over 800 nm. The PEG-ss-COS-ss-PEI copolymers had much lower cytotoxicity and displayed high transfection efficiency than the control branch, indicating that a redox-responsive copolymer composed of low molecular weight PEI, chitosan oligosaccharide and PEG via disulfide-containing linkages can be a useful gene delivery nanocarrier.

#### Enzymes-Responsive Polymers

**FIGURE 5:**
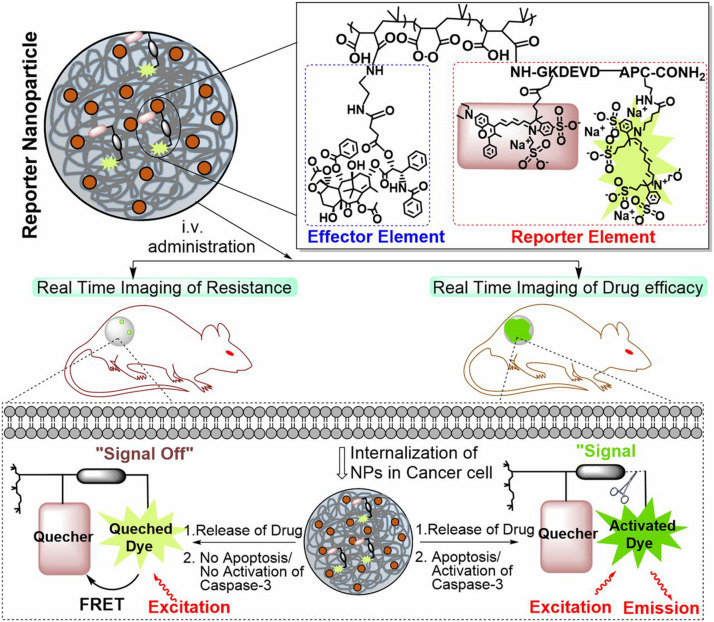
Schematic illustration of the construct of a caspase-3 enzyme-responsive nanoparticle. The reporter nanoparticle comprises three components: a polymeric backbone, an esterase-cleavable prodrug synthesized from an anticancer drug [effector element (EE)], and an activatable reporter element (RE). At the optimal ratio of EE: RE, this stimuli-responsive polymer self-assembles into a nanoparticle. The reporter element is a caspase-3–cleavable sequence consisting of _L_-amino acids GKDEVDAPC-CONH2, to which we conjugated a FRET pair such that cleavage of the DEVD sequence results in removal of the quenching of the fluorescent signal. The effector element is conjugated to the polymeric backbone via an esterasecleavable bond, whereas the reporter element is conjugated via an amide bond with the Gly residue. In normal conditions, the fluorescent signal from the reporter element is in the off-state because the drug is intact inside the nanoparticle. In a drug-sensitive cell (lower right of the schematic), the released drug initiates apoptosis via the activation of the caspase-3 enzyme, which then cleaves the DEVD peptide, unquenching the fluorescent signal (on the state). However, in a non-responder cell (lower left), the failure of the released drug to induce apoptosis means the reporter element remains in the off state. Reprinted from Ashish Kulkarni, Poornima Rao, Siva Natarajan, et al. Reporter nanoparticle monitors its anticancer efficacy in real-time. Proc Natl Acad Sci U S A. 2016;113(15): E2104–13. With permission from reference (Kulkarni A. et al., 2016). FRET, Förster resonance energy transfer; DEVD, Asp-Glu-Val-Asp.

Enzymes serve various functions in all biological and metabolic processes and exhibit abnormal expression levels in many disease-associated microenvironments, especially cancer (Mu et al., 2018). Compared with other stimuli, most enzymic reactions are fast and efficient, and the reaction conditions are moderate. Additionally, most enzyme-responsive nanomaterials, based on polymers, liposomes, small organic molecules, and inorganic/organic hybrid materials, can be triggered with higher specificity, and biocompatibility is beneficial for clinical translation (Mu et al., 2018). So far, several classes of enzymes such as proteases and phosphatases have been regarded as biomarkers for diagnosis and treatment, and many of them have been exploited to generate stimuli-responsive nanomaterials for diagnosis, imaging and drug delivery (He et al., 2016). Among all those enzymes, matrix metalloproteinases (MMPs) are the most well-established ones utilized as stimuli in the enzyme-responsive systems, especially cancer theranostics. MMPs are zinc-dependent endopeptidases responsible for the degradation of extracellular matrixes (ECM) proteins and the modulation of bioactive molecules on the cell surface (Khokha et al., 2013). In cancerous tissue, their expression is much higher than that in normal tissue. They could promote tumor metastases and invasion because of the ability to degrade connective tissue between cells and blood vessels lining, facilitating tumor cells to escape from their original location (Vandenbroucke and Libert, 2014). According to the expression level difference, MMPs have served as triggers, and various nanomaterials have been developed for different purposes (Ansari et al., 2014; Gallo et al., 2014; Wang H.X. et al., 2014; Callmann et al., 2015). For example, Chien et al. developed an enzyme-responsive polymer composed of a hydrophobic backbone and a hydrophilic MMP-responsive peptide (Chien et al., 2013). To date, MMP2 and MMP9 are the most widely explored enzymes for enzyme-responsive drug delivery. Zhu et al. reported a tumor-targeted micellar drug delivery platform prepared by self-assembly of the block copolymers of MMP2-sensitive PEG2000-PTX conjugate, transactivating transcriptional activator peptide-PEG1000-phosphoethanolamine (PE), and PEG1000-PE, acting as MMP2-sensitive functional polymer, cell-penetrating enhancer, and nanocarrier building block, respectively (Zhu et al., 2013). Compared to non-sensitive counterparts, this MMP2 sensitivity of PEG2000-peptide-PTX micelle showed superior cell internalization, cytotoxicity, tumor targeting, and antitumor efficacy, which is promising for effective intracellular drug delivery in cancer therapy. Furthermore, Zhu et al. recently designed another MMP2-sensitive multifunctional polymeric micelle for tumor-targeting co-delivery of siRNA and hydrophobic drugs (Zhu et al., 2014). This micellar nanoplatform was constructed by an MMP2-sensitive copolymer (PEG-pp-PEI-PE) via self-assembly, which displayed exceptional stability, efficient siRNA condensation by PEI, PTX solubilization in the lipid core, and tumor targeting via both the EPR effect and MMP2 sensitivity. Several enzymes can be used as markers to monitor anticancer efficacy. Kulkarni et al. used caspases-3–cleavable sequence as an enzyme reporter element consisting of _L_-amino acids GKDEVDAPC-CONH2 (Kulkarni A. et al., 2016; Figure 5). The effector element is conjugated to the polymeric backbone via an esterase-cleavable bond, whereas the reporter element is conjugated via an amide bond with the Gly residue. In general, the reporter nanoparticles are engineered from a novel two-staged stimuli-responsive polymeric material with an optimal ratio of an enzyme-cleavable drug or immunotherapy (effector elements) and a drug function-activatable reporter element. In a drug-sensitive cell, the loading drug was released due to initiated apoptosis through the activation of the caspase-3 enzyme, which then cleaved the specific peptide, leading to a positive fluorescent signal. However, in a non-responder cell, the process of apoptosis could not be initiated, and the fluorescent signal was silent. This distinction allowed the nanoparticles to monitor the efficacy of treatment and evaluated the tumor resistance to specific anticancer drugs (Kulkarni A. et al., 2016). Although enzyme-responsive polymeric nanomaterials have gained rapid progress and show great therapeutic and diagnostic potentials, especially for cancer at both pre- and clinical levels, challenges remain to be conquered. Different cancers and different stages make the modulation of enzymes difficult. Thus, more effective designing strategies are in need to make the polymeric nanomaterials more precise (Hu et al., 2014; Chandrawati, 2016).

#### Hypoxia-Responsive Polymers

**FIGURE 6:**
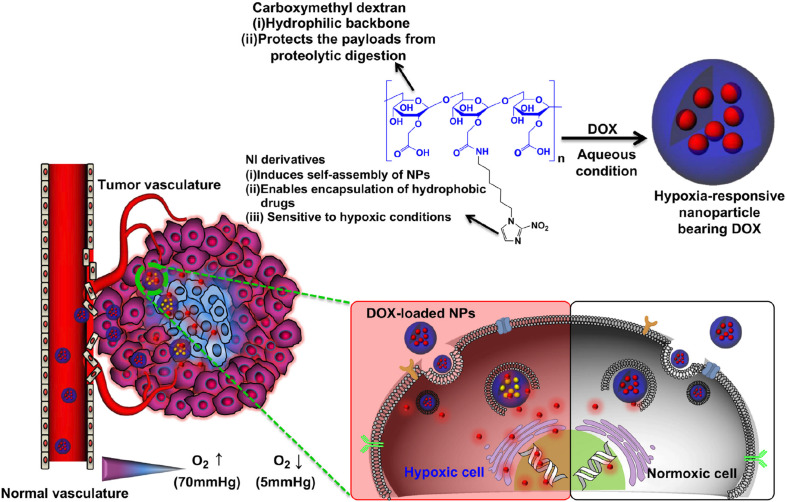
Schematic illustration of a drug-loaded hypoxia-responsive polymer nanoparticle. The hypoxia-responsive polymers can reach the tumor site via the EPR effect, followed by intracellular drug release at hypoxic tissue. Reprinted from Thavasyappan Thambi, V.G. Deepagan, Hong Yeol Yoon, et al. Hypoxia-responsive polymeric nanoparticles for tumor-targeted drug delivery. Biomaterials. 2014;35(5):1735–43. With permission from reference (Thambi et al., 2014). NPs, nanoparticles; EPR, enhanced permeation and retention; DOX, doxorubicin.

Hypoxia is a specific microenvironment involved in the pathogenesis of cancer. Hypoxia-associated pathological state with insufficient oxygen plays an essential role in metastasis and chemotherapy resistance in various kinds of cancers, which provide an opportunity for cancer-specific drug delivery using reduced oxygen partial pressure as a trigger (Brown and Wilson, 2004; Rao et al., 2018). Hydrophobic nitroimidazole is a well-known hypoxia-responsive electron acceptor which can convert into hydrophilic 2-aminoimidazole under hypoxia condition, resulting in the delivery of the loaded DOX from the nanocarrier system to the microenvironment (Uthaman et al., 2018). Thambi et al. developed hypoxia-responsive polymers composed of a 2-nitroimidazole derivative and the backbone of a carboxymethyl dextran (CMD), selectively release drugs under hypoxic conditions (Thambi et al., 2014; Figure 6). The anticancer drug DOX was encapsulated in the polymeric nanoparticles, released at a markedly elevated rate under hypoxic conditions compared with normoxic conditions. In another report, He et al. designed fabrication of dual-sensitive nanoparticles with hypoxia and photo-triggered release of the drug DOX. Dual stimuli nanoparticles were developed through the self-assembly of polyethyleneimine-nitroimidazole micelles (PEI-NI), further co-assembled with Ce6-linked hyaluronic acid (HC), and nitroimidazole was incorporated in the micelles as a hypoxia-responsive electron acceptor that converted to hydrophilic 2-aminoimidazole under hypoxic conditions (He et al., 2018). The Azobenzene group is another hypoxia-sensitive moiety. Kulkarni et al. reported self-assembled polymersomes consisting of poly(lactic acid)–azobenzene–poly(ethylene glycol) and anticancer drugs gemcitabine and erlotinib (Kulkarni P. et al., 2016). This polymeric nanoparticle released the encapsulated anticancer drugs to the pancreatic cancer cells under hypoxic conditions. Biomacromolecules such as siRNA can also be delivered selectively to tumor sites by hypoxia-responsive polymeric nanoparticles. Perche et al. reported hypoxia-induced siRNA delivery using a polymer nanocarrier consisting of PEG, azobenzene, polyethyleneimine, and phospholipid (Perche et al., 2014). The siRNA polymer nanocarriers can be activated to disassemble in oxygen-deprived microenvironments by introducing an azobenzene group between PEG and PEI polymer segments. In the hypoxic environment, the azobenzene bond of the nanoparticles cleaved and deshielded the PEG coating. The responsive polymer nanoparticles with siRNA loading induced efficient gene silencing that mimic the hypoxic tumor microenvironment, representing an ideal hypoxia-responsive nanocarrier for cancer therapy. Although hypoxia-responsive nanoparticles have unique advantages in cancer therapy, it is challenging to deliver polymeric nanoparticles to hypoxic areas because they are commonly far from the vasculatures. Hence, the diffusion rate of the nanoparticles is sometimes insufficient. Thus, polymeric nanocarriers which can release hypoxia-responsive prodrugs to the hypoxic areas should be a better option due to the higher diffusion rates of small molecules.

#### Other Internal Stimuli

In addition to the internal stimuli mentioned above, there are still some other internal ones, such as adenosine triphosphate (ATP) (Lai et al., 2015; Saravanakumar et al., 2017). To conclude, the internal stimuli can increase the accumulation of polymer-based nanoparticles and facilitate drug delivery in targeting tissues because of the changes in pathophysiological properties.

### External Stimuli

#### Light-Triggered Polymers

**FIGURE 7:**
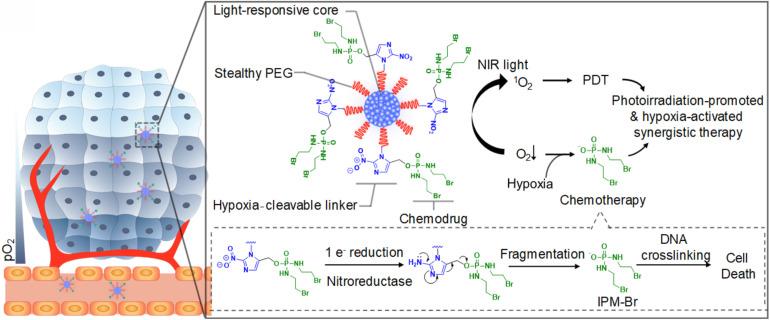
Schematic illustration of the light-responsive SPNpd for synergistic cancer therapy. The nano-prodrug is assembled from an amphiphilic semiconducting brush polymer grafted with chemo drug side chains through a hypoxia-cleavable linker. It has three critical units: the light-responsive photodynamic SPN core, hypoxia-cleavable linker and the chemotherapeutic drug, a bromoisophosphoramide mustard intermediate (IPM-Br). Upon the photoirradiation at 808 nm, this organic photodynamic nano-prodrug (SPNpd) can specifically release the chemodrug under photoirradiation-promoted hypoxia tumor microenvironment to exert synergetic PDT and chemotherapy. Reprinted from Dong Cui, Jiaguo Huang, Xu Zhen, et al. Semiconducting Polymer Nano-prodrug for Hypoxia-activated Synergetic Photodynamic Cancer therapy. Angew Chem Int Ed Engl 2019;58(18):5920–24. With permission from reference (Cui et al., 2019). SPNpd, semiconducting polymer nanoprodrug; SPN, semiconducting polymer nanoparticle; PDT, photodynamic therapy.

Among all external stimuli, light is the most commonly exploited one due to the ease of control and utilization (Zhang et al., 2019). The light-responsive polymeric nanomaterials have been widely applied for cancer therapy, mainly photothermal therapy (PTT) and photodynamic therapy (PDT). PTT refers to the use the light-sensitive materials that can convert the light energy to heat to increase the temperature and trigger the death of the surrounding cancer cells (Kim et al., 2016; Liu et al., 2019). Compared with other therapies, PTT allows the precise dosage of external irradiation to diminish the side-effect of the surrounding tissues. Furthermore, studies have shown that PTT is highly effective for various cancer and has multiple functions in treatment (Liu et al., 2019). PTT can combine with other therapies such as surgery (Wang S. et al., 2018), chemotherapy (Liu T. et al., 2014), radiotherapy (Yong et al., 2015), immunotherapy (Wang C. et al., 2014) to improve the overall treatment results and benefit from the outcomes or effects. So far, many nanomaterials such as semiconducting polymers have been explored for PTT, and some of them have been under clinical investigation for tumors such as head and neck cancers and primary/metastatic lung cancers (Shi et al., 2017; Liu et al., 2019). For example, Cao et al. reported a light-breakable amphiphilic block copolymer micelle with a NIR dye cypionate (Ex/Em: 780/808 nm) encapsulated into the hydrophobic core (Cao et al., 2013). A dual NIR emission induced a faster photocleavage reaction when irradiated by NIR light (765 nm), which facilitated the faster dissociation of the micelles under NIR illumination ablate the tumor tissues *in vivo* through PTT. Bagheri et al. developed an *in situ*, one-pot polymerization-induced self-assembly method to synthesize light-responsive pyrene-containing nanoparticles (Bagheri et al., 2019). Cleavage of the pyrene moieties triggered a hydrophobic-to-hydrophilic transition of the core-forming block and the dissociation of the nanoparticles, and PTT triggered the therapeutic compounds to release into the tumor. PDT is another important application for light-responsive nanomaterials and has emerged as a precise treatment modality. It utilizes the photosensitizers, which can be activated by the light of a certain wavelength, to generate cytotoxic ROS that can oxidize key cellular macromolecules and induce tumor cell ablation (Lucky et al., 2015). Nanoparticles utilized in PDT can serve as the carriers of photosensitizers or the energy transducers themselves. Polymers capable of encapsulating the photosensitizers can target the tumor sites and release the payloads to generate ROS (Synatschke et al., 2014). Similar to PTT, PDT also can combine with other therapies or improve the overall outcomes (Yu et al., 2015; Wang et al., 2016a, 2017). For example, Cui and co-workers synthesized a semiconducting polymer nanoprodrug (SPNpd) that can specifically release the chemo drugs under a photoirradiation-promoted hypoxic environment to exert synergetic PDT and chemotherapy (Cui et al., 2019; Figure 7). SPNpd is self-assembled from an amphiphilic polymer brush comprising a light-responsive photodynamic backbone grafted with PEG and conjugated with the chemodrug molecules via hypoxia-cleavable linkers. SPNpd (30 nm) enabled effective accumulation to the target site of breast cancer xenograft and possessed a synergistic photodynamic efficacy and chemotherapy, which acted as a promising photoirradiation-promoted and hypoxia-responsive polymeric nanoprodrug system for cancer therapy.

Light responsive polymeric nanomaterials can also be used for photo-triggered drug release when illuminated by external light. The mechanisms generally include photo-induced chemical effects, decreased hydrophobicity, and photothermal effect (Shim et al., 2017; Son et al., 2019). These strategies allow the nanomaterials to release therapeutic agents at the target sites upon the external light. For example, Cao et al. developed the biocompatible diblock copolymer micelles for controlled drug delivery. Upon the NIR irradiation, the NIR-sensitive hydrophobic core could increase the polarity and destabilize the micelles, leading to a shifted hydrophilic–hydrophobic balance which could control the release of loading drugs (Cao et al., 2016). Bagheri et al. developed a drug delivery system using NIR light and upconversion nanoparticles (UCNPs), emphasizing the use of photo-responsive compounds and polymeric materials conjugated onto UCNPs. This drug delivery system can be activated by low-intensity NIR illumination; thus, it is highly desirable to avoid exposing living tissues to excessive heat and reduce the *in vivo* application of this polymeric nanomaterials (Bagheri et al., 2016).

#### Temperature-Responsive Polymers

**FIGURE 8:**
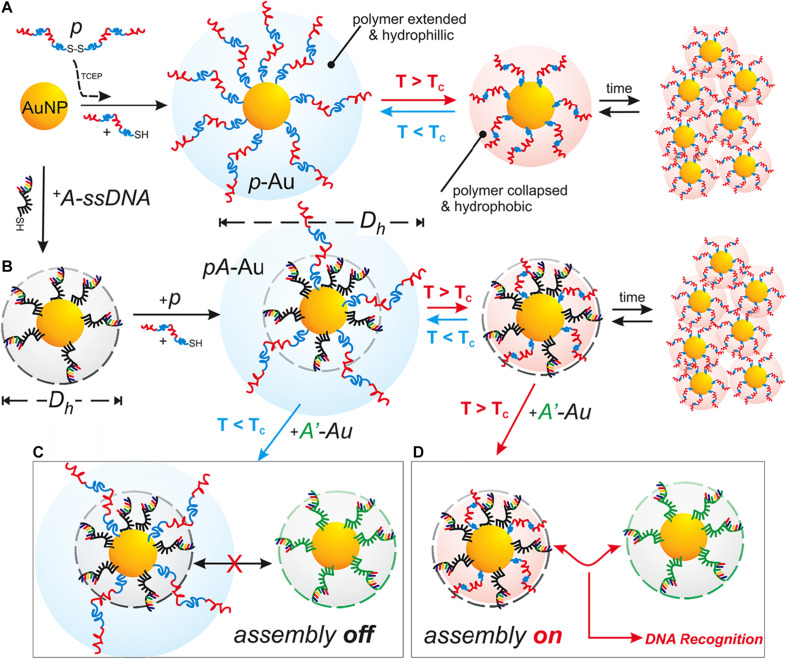
Schematic Illustration of the temperature-responsive polymer systems. **(A)** The Au NPs were functionalized with freshly reduced pNIPAAm-*co*-pAAm copolymer (*p*), and the thermal response-based aggregation was measured. **(B)** The Au NPs were first functionalized with thiolated *A*-type ssDNA, then co-functionalized with *p*. The assembly of *Ap*-Au with complementary *A’*-Au was then blocked at *T* < *T*_*C*_ **(C)**, but promoted at *T* > *T*_*C*_ **(D)**. Reprinted from Hamner KL, Alexander CM, Coopersmith K, et al. Using temperature-sensitive smart polymers to regulate DNA-mediated nanoassembly and encoded nanocarrier drug release. ACS Nano. 2013;7(8):7011–20. With permission from reference (Hamner et al., 2013). Au NPs, gold nanoparticles; *Tc*, critical temperature.

Temperature is another commonly utilized external stimulus to trigger the thermo-sensitive nanomaterials to release the loading agents. The temperature-responsive polymers can respond to the temperature changes and switch their structure or the aqueous solubility. Thus, the encapsulated drugs could be released at the target tissues (Karimi et al., 2016). The polymer undergoes a reversible change of phase at the specific temperature, called lower critical solution temperature (LCST) or upper critical solution temperature (UCST). The therapeutic agents can be easily encapsulated into the polymers at LCST and released at the targeting sites upon the external temperature changes (Bikram and West, 2008). Poly(N-isopropyl acrylamide) (PNIPAM) and its derivatives have been widely investigated because of the attractive LCST, which is close to the physiological temperature of the human body. The LCST of PNIPAM is around 32°C, and by coupling other materials (e.g., polymers, liposomes, proteins), the LCST could be optimized to control the drug release (Bikram and West, 2008; Karimi et al., 2016). For example, Kakwere et al. developed the nanohybrids by incorporating cubic-IONPs within a thermo-responsive polymer shell composed of PNIPAM/PEGA. The LCST of these nanohybrids was about 37°C. The phase transition may occur, leading to the release of the loading drugs upon the temperature changes (Kakwere et al., 2015). In another study, Neradovic et al. developed block copolymers of PEG as a hydrophilic block and poly(N-isopropyl acrylamide) (PNIPAAm) or poly(NIPAAm-co-N-(2-hydroxypropyl) methacrylamide-dilactate) [poly(NIPAAm-co-HPMAm-dilactate)] as the thermosensitive block that could self-assemble into nanoparticles (Neradovic et al., 2004). These copolymers formed a novel type of thermosensitive micelle, and the micelles were destabilized to release their cargo at temperatures above the LCST of 37°C with a triggered drug release profile. Qin et al. used poly(ethylene oxide)-block-poly(N-isopropyl acrylamide) (PEO-bpNIPAm) block copolymers to generate polymer micelles which became amphiphilic in water above 37°C and self-assemble into micelles encapsulating both hydrophilic and hydrophobic molecules (Qin et al., 2006). When the temperature is decreased, however, the micelles disassemble and release the molecules triggered by temperature. In addition, temperature-responsive polymeric nanoplatforms can also be used to combine externally heat-triggered treatment and localized chemotherapy under magnetic hyperthermia (MHT) conditions and may target heat more specifically and boost the drug release on demand. Mai and co-workers have engineered magnetic thermo-responsive iron oxide nanocubes (TR-cubes) to merge MH treatment with heat-mediated drug delivery (Mai et al., 2019). IONPs with a cubic shape showed remarkable heat performance under MHT conditions, and these TR-cubes can carry chemotherapeutic doxorubicin (DOX-loaded-TR-cubes) without compromising their thermo-responsiveness. A uniform and thick polymer shell on each nanocube enabled the thermo-responsive polymer nanosystem to combine MH and heat-mediated drug delivery, making the dual MH/heat-mediated chemotherapy possible. Furthermore, the temperature-responsive polymeric nanocarriers are also effective for delivery of genes. For example, Hamner et al. used a DNA-capped thermosensitive copolymer for chemotherapy drug DOX delivery (Hamner et al., 2013; Figure 8). They synthesized a thermoresponsive pNIPAAm-co-pAAm polymer to regulate DNA interactions in both a DNA-mediated assembly system and a DNA-encoded drug delivery system. The temperature-responsive behavior of the polymer regulated the accessibility of the sequence-specific hybridization between complementary DNA-functionalized gold nanoparticles, with a transition temperature (*T*_*C*_) of 51°C. The LCST smart polymer was shown to decrease drug release kinetics and equilibrium at *T* < *T*_*C*_, but increase release at *T* > *T*_*C*_, thus allowing for a successful improvement of the drug delivery. In another study, Li et al. reported a rod-shaped ternary polyplex micelle via complexation between the mixed block copolymers of PEG-b-poly [PEG-b-PAsp(DET)] and poly(N-isopropylacrylamide)-b-PAsp(DET) [PNIPAM-b-PAsp(DET)] and plasmid DNA at room temperature, which exhibited unique temperature-responsive formation of a hydrophobic intermediate layer between PEG shells and plasmid DNA cores through facile temperature increase from room temperature to body temperature (∼37°C) (Li et al., 2015). This temperature-responsive micelle system possessed great potentials as efficient systemic non-viral gene delivery systems.

#### Magnetic Field-Responsive Polymers

**FIGURE 9:**
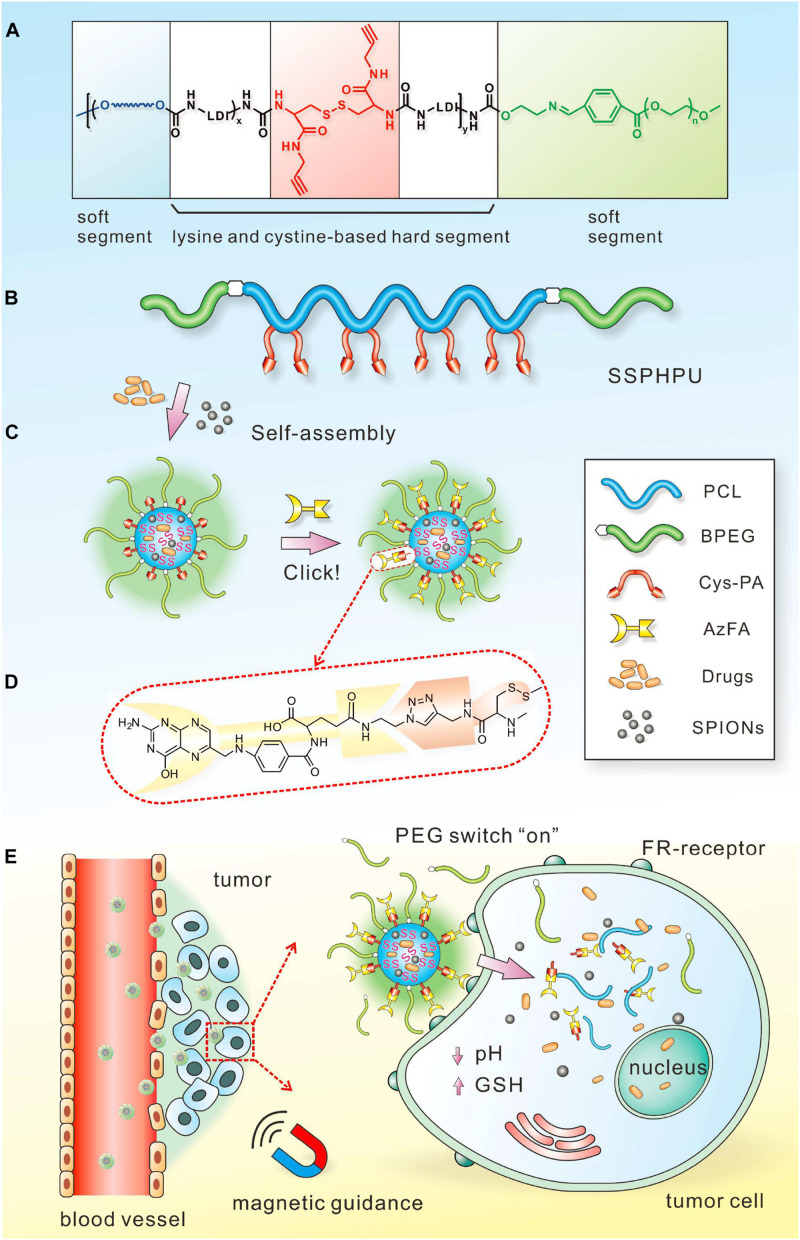
Schematic illustration of the magnetic field-responsive polymer micelles. Schematic chemical structure **(A)** and molecular architecture **(B)** of clickable multiblock polyurethanes (MPUs). **(C)** Self-assembly of MPU micelles and post-conjugation of folic acid via click chemistry. **(D)** Schematic illustration of FA residues on the interface of polymer micelles. **(E)** Illustration of magnetic-guided and PEG-switched targeting and release properties of MPU nanocarriers. Reprinted from Jing Wei, Xiaoyu Shuai, Rui Wang, et al. Clickable and imageable multiblock polymer micelles with magnetically guided and PEG-switched targeting and release property for precise tumor theranostics. Biomaterials. 2017;145:138–53. With permission from reference (Wei et al., 2017).

The magnetic field can serve as an external stimulus for cancer therapy by controlling the drug release of the polymeric nanomaterials. Magnetic field-responsive polymers, typically incorporating the therapeutic components and magnetic nanoparticles, can produce heat in the presence of alternating magnetic fields (AMF), and MHT is an effective therapy method used for cancers (Chen et al., 2015; Zhou et al., 2018). For example, Le et al. synthesized IONPs coated with a polycationic polymer poly-L-lysine (PLL) to prevent their aggregation and enable their administration, which exhibited superior anticancer efficacy in the magnetic hyperthermia treatment of glioblastoma (Le Fevre et al., 2017). Jaidev et al. developed tumor-targeted fluorescent IONPs and gemcitabine encapsulated poly(lactide-co-glycolide) (PLGA) nanospheres conjugated with human epidermal growth factor receptor antibodies for magnetic hyperthermia of pancreatic cancer. The nanoparticles with surface modification of polymeric nanocarriers for antibody binding could enhance tumor retention through active targeting, and their multifunctional abilities significantly inhibited tumor growth *in vivo* (Jaidev et al., 2017). Compared with PTT, MHT can overcome the limitations of tissue penetration and provide an invasive method for cancer therapy. Additionally, MHT has progressed in clinical trials for different cancers, including prostate cancer, oral cancer, glioma, esophageal cancer, and so forth (Johannsen et al., 2005). Additionally, magnetic-sensitive polymeric nanoparticles can control drug delivery through the heat energy produced by AMF. In a recent study, Wei et al. designed a responsive polymeric platform with a clickable and imageable nano vehicle assembled from multiblock polyurethanes (MPUs) for precise tumor diagnosis and treatment (Wei et al., 2017; Figure 9). The soft segments of the polymers are based on detachable PEG and degradable PCL, and the hard segments are constructed from lysine- and cystine-derivatives bearing reduction-responsive disulfide linkages and click-active alkynyl moieties, allowing for post-conjugation of targeting ligands via click chemistry. They found that the cleavage of PEG corona bearing a pH-sensitive benzoic-imine linkage could act as an on-off switch, which can activate the clicked targeting ligands under extracellular acidic microenvironment, followed by triggering the core degradation and payload release in the tumor cells. Moreover, in combination with superparamagnetic IONPs entrapped in the micellar core, the prepared micelles present excellent MRI contrast effects and T2 relaxation *in vitro* and magnetically guided MRI multimodal targeting therapeutics to tumor resulting in precise anticancer therapy and specifically enhanced MR imaging. Lee et al. developed the pluronic/polyethyleneimine shell crosslinked nanocapsules entrapping magnetite nanocrystals (PMCs) that could deliver siRNA and enhance the intracellular uptake upon exposure to a magnet (Lee et al., 2010). Although only *in vitro* experiments were conducted in this study and the effect of magnetic force for triggered release still needs additional *in vivo* tests, this study provided a novel polymer-based nanoplatform for magnetically triggered delivery of negatively charged therapeutic agents, as well as for diagnostic MRI.

#### Other External Stimuli

Some other external stimuli also have great promises for cancer theranostics, such as ultrasound (Paris et al., 2015; Jin et al., 2017), radiation (Fan et al., 2015; Liu et al., 2017), radiofrequency (Rejinold et al., 2015; Liu et al., 2018), and electric field (Ge et al., 2012; Kolosnjaj-Tabi et al., 2019). The corresponding polymeric nanomaterials have different properties, which can combine with the external stimuli to realize different requirements, including trigger the release of loading drugs, kill the cancer cells through different mechanisms, enhance the anticancer efficacy with another treatment method, and fulfill cancer imaging/detection/diagnosis (Fleige et al., 2012; Sadhukha et al., 2013; Chen et al., 2015; Mohamed et al., 2019).

### Dual/Multi-Stimuli Responsive Polymers

Every kind of stimuli-responsive polymeric nanomaterials has its limitations. For instance, the internal stimuli, such as pH, redox potential, enzymes induced by the pathophysiological property between cancerous and normal tissues undergo dynamical changes affected by multiple factors *in vivo*. Thus, it is not easy to control these nanomaterials precisely, and the speed of response *in vivo* could limit their usage. As for the external stimuli, the key points are supposed to focus on how to increase the tissue penetration for the deep localized tumors and minimize the damage of the surrounding normal tissues with a maximized specificity and selectivity. In recent years, dual/multi-stimuli responsive polymeric nanomaterials have been generated for cancer theranostics, which can combine the advantages of each kind of materials and overcome the limitations of single-stimulus (Cheng et al., 2013; Fu et al., 2018). Dual-stimuli responsive polymeric nanoparticles have been developed that respond to a combination of two signals such as pH/redox, pH/magnetic field, pH/temperature, double pH, temperature/reduction, temperature/enzyme, temperature/magnetic field, and so on. Multi-stimuli responsive polymeric nanoparticles have been developed that respond to more signals such as temperature/pH/redox, temperature/pH/magnetic, pH/redox/magnetic, temperature/redox/guest molecules, temperature/pH/guest molecules, and so on. To date, the majority of multi-stimuli-responsive polymer nanoparticles are based on pH responsiveness due to the significant pH variations between the acidic tumor microenvironments and the normal tissues.

**FIGURE 10:**
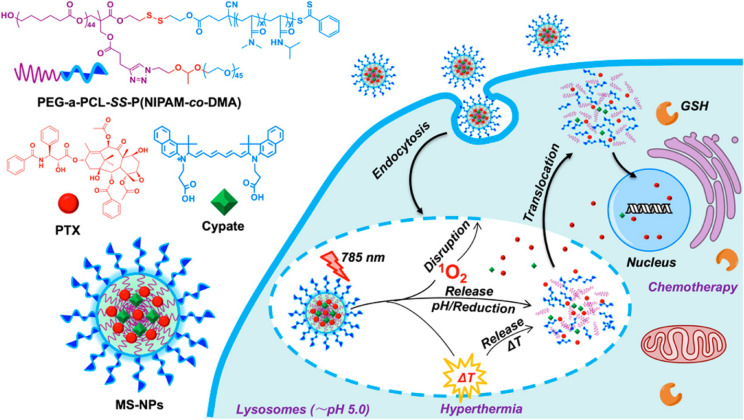
Schematic illustration of NIR light/pH/reduction-responsive nanoparticles. The NIR Light/pH/Reduction–responsive nanoparticles consist of PEG-a-PCL-SSP(NIPAM-co-DMA) (S1) star quaterpolymer for precise cancer therapy synergistic effects. Reprinted from Xiaonan An, Aijun Zhu, Huanhuan Luo, et al. Rational Design of Multi-Stimuli-Responsive Nanoparticles Precise Cancer Therapy. ACS Nano. 2016;10(6):5947–58. With permission from reference (An et al., 2016). NIR, near-infrared; PEG, poly(ethylene glycol); PCL, poly(ε-caprolactone); NIPAM, N-isopropylacrylamide; DMA, dimethylacrylamide; GSH, cytoplasmic glutathione; MS-NPs, multi-stimuliresponsive nanoparticles.

For example, Li et al. designed a transformable polymer nanoparticle system with pH and Light dual-stimuli (Li et al., 2017a). The pH/Light dual-stimuli polymer nanoparticles accumulated in the tumor sites based on the EPR effect, the sheddable modifications on the nanoparticles were stripped in the trigger of acidic pH. Then TAT peptides were exposed, causing improved cell association and internalization. IR-780 light irradiation promoted the DOX release loaded in the nanoparticles, leading to the death of tumor cells. Li et al. constructed a polymer nanoparticle with tumor-specific pH-responsive activation and H_2_O_2_ induced self-destruction based on optimized block copolymer, PEG-b-P(PBEM-co-PEM), for efficient *in vivo* antitumor application (Li et al., 2017b). The novel glucose oxidase-loaded therapeutic polymeric nanoreactors efficiently kill tumor cells and eliminate tumor via the synergistic effect. Furthermore, a block copolymer prodrug-based polymersome nanoreactor was constructed by Li and co-workers that can be specifically activated by acidic pH at the tumor site and produce H_2_O_2_ to further trigger the rapid release of camptothecin, which can achieve orchestrated oxidation/chemo-therapy of cancer via specific activation of increased tumor oxidative stress and higher released camptothecin drugs for cancer therapy (Li et al., 2017c). In another study, An et al. synthesized a star quaterpolymer with suitable LCST (44.7°C) and cleavable acetal and disulfide moieties assembled into the NIR light/pH/reduction-responsive nanoparticles (An et al., 2016; Figure 10). The multi-stimuli-responsive nanoparticles with a NIR photothermal agent and chemotherapeutic compound can exhibit smart drug release in response to intrinsic pH and reduction stimuli and can be further boosted by NIR light irradiation. The NIR light/pH/reduction-responsive nanoparticles also exhibited enhanced tumor accumulation and intracellular drug translocation in cancer cells, which synergized the photo-induced thermo-chemotherapeutic efficacy with anticancer efficiency. The dual/multi-stimuli responsive nanoparticles are highly desired for biomedical applications, especially drug delivery in cancer therapy. However, there are also several limitations, such as the low drug loading capacity, insufficient biocompatibility, *etc*. Future research should devote the effort to increase the loading efficiency and improve the biocompatibility and degradability of dual/multi stimuli-responsive polymeric nanomaterials for cancer therapy.

## Clinical Studies of Polymeric Delivery Systems

Over the last two decades, polymer-based nanoplatforms have been extensively applied for various medical applications and human studies (Hua et al., 2018). Polymer-based nanoplatforms and liposomes are the most clinically available nanomaterials for human use and have been evaluated for therapeutic delivery in cancer therapy (De Jong and Borm, 2008; Majumder and Minko, 2021). Because of the unique features of polymer-containing nanodrugs in prolonging circulating half-life and improving passive tumor targeting by increasing the size of a drug, rapid progress has been made on developing polymeric nanosystems for targeted therapeutic delivery and diagnostic applications. As reported, some polymer micelles are already available for clinical use, and some polymer-drug conjugates and nanospheres are under clinical development in cancer treatment (Luque-Michel et al., 2017).

Many polymer-containing nanodrugs are being investigated in clinical trials due to the broad applicability of polymer-based nanoformulations (Ventola, 2017). For example, Opaxio (Xyotax) is a nano drug-containing polyglutamic acid-conjugated (poliglumex) paclitaxel, and early stage trials of Opaxio in patients with ovarian cancer and fallopian tube cancers showed promising clinical results (Caster et al., 2017). Furthermore, the ongoing phase III trial of Opaxio as maintenance therapy for ovarian cancer patients obtained complete responses after taxane and platinum therapy. CRLX-101, drug–conjugate formulation of camptothecin and a cyclodextrin-PEG polymer, has shown promising early therapeutic profiles in phase I/II clinical trials in patients with solid tumors such as lung cancers (SCLC and NSCLC) and gynecological malignancies (Weiss et al., 2013; Caster et al., 2017). CRLX-301(NCT02380677) is another docetaxel-conjugate polymer, which has been studied in a phase I/IIa clinical trial in the treatment of advanced solid tumors. In addition, NK012 is a polymeric formulation of SN-38 (an active metabolite of the topoisomerase inhibitor irinotecan), and two phase I trials and several phase II trials utilizing this micellar nanoformulation of SN-38 have been completed or still are ongoing in solid tumors including NSCLC (Caster et al., 2017) and triple-negative breast cancer (NCT00951054). Genexol-PM, a MPEG-block-D, L-PLA micellar formulation of paclitaxel, is being developed as alternative Cremophor-based paclitaxel. Recently, Genexol-PM is extensively investigated in phase I/II clinical trials in various countries, approved for treating metastatic breast cancer and advanced lung cancer in South Korea (Havel, 2016). Several phase II trials in solid tumors of metastatic breast cancer and NSCLC have shown a low rate of toxic reactions and a favorable rate of overall remission (Lee et al., 2008; Ahn et al., 2014). A summary of clinical studies of polymeric delivery systems for cancer therapy is presented in Table 2 (Valle et al., 2011; Cabral and Kataoka, 2014). More clinical trials are being conducted, and novel techniques are being developed to reduce the toxicity issues and safe use of polymer-based nanomedicines in human health.

**TABLE 2:** Clinical studies of polymeric delivery systems for cancer therapy.

Name	Polymer	Drug	Indication	Clinical status
Genexol-PM^®^	mPEG-PLA	Paclitaxel	◆ Recurrent breast cancer ◆ Unresectable locally advanced or metastatic pancreatic cancer ◆ Advanced Urothelial Cancer	◆ Phase IV (NCT00912639) ◆ Phase II (NCT00111904) ◆ Phase II (NCT01426126)
Docetaxel-PM	mPEG-PLA	Docetaxel	◆ Head and Neck Squamous Cell Carcinoma	Phase II (NCT02639858)
NK105	PEG-modified poly(α,β-Asp)	Paclitaxel	◆ Recurrent or metastatic breast cancer	◆ Phase III (NCT01644890)
NC-4016	mPEG-PGA	Oxaliplatin	◆ Advanced solid tumors or lymphoma	◆ Phase I (NCT01999491)
Cripecdocetaxel	Thermosensitive PEG-β-poly(N-(2-hydroxypropyl)-methacryla-mide-lactate)	Docetaxel	◆ Cancer, Solid tumors	◆ Phase I (NCT02442531)
NK012	PEG modified PGA	SN38	◆ Triple negative breast cancer ◆ Refractory solid tumors ◆ Metastatic colorectal cancer in combination with 5-fluorouracil	◆ Phase II (NCT00951054) ◆ Phase I (NCT00542958) ◆ Phase II (NCT01238939)
SPI-77	PEG	Cisplatin	◆ Ovarian tumor ◆ Osteosarcoma Metastatic	◆ Phase II (NCT00004083) Phase II (NCT00102531)
NC-6004	PEG-PGlu	Cisplatin	◆ Recurrent or Metastatic Squamous Cell Carcinoma of the Head and Neck	◆ Phase I/II (NCT03109158)
CT-2106	Poly(L-glutamic acid)	Camptothecin	◆ Ovarian Cancer ◆ Colorectal Cancer ◆ Unspecified Adult Solid Tumor	◆ Phase II(NCT00291837) ◆ Phase I/II(NCT00291785) ◆ Phase I (NCT00059917)
EZN-2208	4-arm PEG	SN38	◆ Advanced Solid Tumors, Lymphoma	◆ Phase I (NCT00520637)
NKTR-102	4-arm PEG	Irinotecan	◆ Advanced Cancer, Metastatic Solid Tumors ◆ Metastatic and recurrent NSCL	◆ Phase I (NCT01976143) ◆ Phase II (NCT01773109)
XYOTAX (CT-2103) Paclitaxel	Poly(L-glutamic acid)	Paclitaxel	◆ Glioblastoma Multiforme, Non-small Cell ◆ Lung Cancer	◆ Phase II (NCT01402063) ◆ Phase II (NCT00487669)
NK911	PEGpoly(α,β-Asp)	Doxorubicin	◆ Metastatic pancreatic cancer	◆ Phase II (Cabral et al., 2014)
SP1049C	Pluronic^®^ P-61 and F-127 block copolymers	Doxorubicin	◆ Advanced refractory adenocarcinoma of the esophagus or GEJ	◆ Phase II (Valle et al., 2011)
NKTR-105	4-arm PEG	Docetaxel	◆ Metastatic or locally recurrent breast cancer	◆ Phase III (NCT01492101)
XMT-1001	PHF (Succinamidoester)	Camptothecin	◆ Advanced solid tumors	◆ Phase I (NCT00455052)
Doxorubicin Transdrug (Livatag)	PIHCA	Doxorubicin	◆ Advanced hepatocellular carcinoma	◆ Phase III (NCT01655693)
CRLX301	Cyclodextrin-PEG	Docetaxel	◆ Advanced solid tumors	◆ Phase I/IIa (NCT02380677)

*Asp, aspartic acid; PEG, poly(ethylene glycol); GEJ, gastroesophageal junction; mPEG, methoxypoly(ethylene glycol); NCT#, ClinicalTrials.gov registry number; PGA, poly(L-glutamic acid); PLA, poly (D,L lactic acid); PHF, poly(1-hydroxyl-methylethylene hydroxyl-methyl-formal); PIHCA, poly(isohexyl cyanoacrylate).*

## Conclusion and Perspective

In summary, this review introduces representative theranostic polymeric nanomaterials and their advantages and disadvantages in the practical use as well as their unique properties. In particular, recent advances of stimuli-responsive polymeric nanocarriers in the development of drug delivery are discussed in cancer therapy, where stimuli-responsive polymeric nanocarriers have been shown to own the possibility of controlled release of drugs/genes at the target sites by acting as an active participant rather than passive mediators. Various studies on stimuli-responsive polymers have been published, which showed that multifunctional polymeric nanosystems are promising to be effective platforms for drug/gene delivery in response to a range of internal (pH, redox potential, enzymes, hypoxia, *etc.*) and external stimuli (light, magnetic field, ultrasound, temperature, radiation, *etc.*). The internal stimuli-responsive polymeric nanosystem relies on the abnormal microenvironments in various cancers, such as acidic extracellular pH and hypoxia, for targeted drug delivery, while the external stimuli-responsive nanosystem requires prior information on the target-specific site for efficient therapy. Moreover, studies have shown that the applications of polymeric nanomaterials in various cancers have achieved positive effects in both diagnosis and treatment monitoring, including enhanced therapeutic outcomes and reduced systemic side effects compared to traditional anticancer drugs.

Despite various advantages of stimuli-responsive polymeric nanomaterials over conventional therapies, we should know that they are not perfect and many crucial issues and challenges still remain to be addressed. At first, there are a number of biological components that polymeric nanomaterials would encounter after *in vivo* administration, including biological molecules, cells, and tissues/organs. The features of polymeric nanomaterials, such as surface charge and size, will determine the subsequent biodistribution and cellular responses of polymeric nanomaterials. Secondly, it is essential to improve the stimuli sensitivity of polymeric nanomaterials in target sites because non-specific distribution of stimuli can lead to off-target effects. For example, low pH can also be found in some normal tissues, so the degree of acidic pH to which polymeric nanomaterials would respond may play an important role in determining the release amount and release rate to the target sites. Moreover, the heterogeneity of tumor types and stages greatly influence the status of internal stimuli, which should be examined extensively before the synthesis of polymeric nanomaterials. Thirdly, the current polymeric nanomaterials still have limitations for clinical or practical use due their complicated design, limited biostability *in vivo*, and potential toxicity.

We have given several examples published for the commonly used multifunctional and stimuli-responsive polymeric nanomaterials in different cancers and their roles in the process of treatment. However, polymeric nanomaterials for only imaging or only therapy are not included because of the topic request. Importantly, the future trend for polymeric nanomedicine should focus on combinational therapy, which refers to the combination of nanomedicine and gene therapy or immunotherapy for the improved efficacy of 1 + 1 > 2. Moreover, with the aging population increasing worldwide, cancers are a severe threat to people’s health. Therefore, a better understanding of the physiological microenvironments of cancers and the further development of polymeric nanocarrier-based drug systems are necessary for targeted therapeutic delivery applications. More attention should be paid to the progress of different cancers and what stimuli-responsive polymeric nanomedicine can do to globally reduce the social burden and contribute to the medical field.

## Author Contributions

DC and YM wrote the manuscript and developed the tables. DC and XX did the literature research. DC and JX developed or collected the figures. JX and SJ reviewed, edited, and supervised. All authors contributed to the article and approved the submitted version.

## Conflict of Interest

The authors declare that the research was conducted in the absence of any commercial or financial relationships that could be construed as a potential conflict of interest.

## Abbreviations

DOX: doxorubicin
PEG: polyethylene glycol
MRI: magnetic resonance imaging
IONPs: iron oxide nanoparticles
QDs: quantum dots
AuNPs: gold nanoparticles
CT: computed tomography
CNTs: carbon nanotubes
SWCNTs: single-walled carbon nanotubes
MWCNTs: multi-walled carbon nanotubes
EPR: permeability and retention
HPMA: N-(2-Hydroxypropyl) methacrylamide
PLGA: poly (lactic co-glycolic acid)
NIR: near infrared
HBPs: hyperbranched polymers
PLA: poly (lactic acid)
PGA: poly glycolic acid
Gd: gadolinium
PDT: photodynamic therapy
PEI: polyethylene imine
CHT: chemotherapy
RT: radiation therapy
TME: tumor microenvironment
PEO: poly(ethylene oxide)
PCL: poly( ε-caprolactone )
GSH: glutathione tripeptide ( γ-glutamyl -cysteinyl-glycine)
ROS: reactive oxygen species
MDR: multidrug resistance
MMPs: matrix metalloproteinases
ECM: extracellular matrixes
CMD: carboxymethyl dextran
PTT: photothermal therapy
SPNpd: semiconducting polymer nanoprodrug
UCNPs: upconversion nanoparticles
QDs: quantum dots
LCST: lower critical solution temperature
UCST: upper critical solution temperature
MHT: magnetic hyperthermia
TR-cubes: thermo-responsive iron oxide nanocubes
AMF: alternating magnetic field
PLL: polymer poly-L-lysine
MPUs: multiblock polyurethanes
siRNA: small interfering RNA
NSCLC: non-small cell lung cancer.

## References

[B1] Ahn H. K. Jung M. Sym S. J. Shin D. B. Kang S. M. Kyung S. Y. (2014). A phase II trial of Cremorphor EL-free paclitaxel (Genexol-PM) and gemcitabine in patients with advanced non-small cell lung cancer. *Cancer Chemother. Pharmacol.* 74 277–282. 10.1007/s00280-014-2498-5 24906423 PMC4112044

[B2] Alfarouk K. O. Muddathir A. K. Shayoub M. E. (2011). Tumor acidity as evolutionary spite. *Cancers* 3 408–414. 10.3390/cancers3010408 24310355 PMC3756368

[B3] Al-Jamal W. T. Kostarelos K. (2011). Liposomes: from a clinically established drug delivery system to a nanoparticle platform for theranostic nanomedicine. *Acc. Chem. Res.* 44 1094–1104. 10.1021/ar200105p 21812415

[B4] Alsehli M. (2020). Polymeric nanocarriers as stimuli-responsive systems for targeted tumor (cancer) therapy: recent advances in drug delivery. *Saudi Pharm. J.* 28 255–265. 10.1016/j.jsps.2020.01.004 32194326 PMC7078546

[B5] Amin M. L. Joo J. Y. Yi D. K. An S. S. (2015). Surface modification and local orientations of surface molecules in nanotherapeutics. *J. Control Release* 207 131–142. 10.1016/j.jconrel.2015.04.017 25883030

[B6] An X. Zhu A. Luo H. Ke H. Chen H. Zhao Y. (2016). Rational design of multi-stimuli-responsive nanoparticles for precise cancer therapy. *ACS Nano* 10 5947–5958. 10.1021/acsnano.6b01296 27285378

[B7] Ansari C. Tikhomirov G. A. Hong S. H. Falconer R. A. Loadman P. M. Gill J. H. (2014). Development of novel tumor-targeted theranostic nanoparticles activated by membrane-type matrix metalloproteinases for combined cancer magnetic resonance imaging and therapy. *Small* 10 566–575. 10.1002/smll.201301456 24038954 PMC3946335

[B8] Bagheri A. Arandiyan H. Boyer C. Lim M. (2016). Lanthanide-doped upconversion nanoparticles: emerging intelligent light-activated drug delivery systems. *Adv. Sci.* 3:1500437. 10.1002/advs.201500437 27818904 PMC5069703

[B9] Bagheri A. Boyer C. Lim M. (2019). Synthesis of light-responsive pyrene-based polymer nanoparticles via polymerization-induced self-assembly. *Macromol. Rapid Commun.* 40:e1800510. 10.1002/marc.201800510 30176080

[B10] Bejarano L. Jordao M. J. C. Joyce J. A. (2021). Therapeutic targeting of the tumor microenvironment. *Cancer Discov.* 11 933–959. 10.1158/2159-8290.CD-20-1808 33811125

[B11] Bertrand N. Wu J. Xu X. Kamaly N. Farokhzad O. C. (2014). Cancer nanotechnology: the impact of passive and active targeting in the era of modern cancer biology. *Adv. Drug Deliv. Rev.* 66 2–25. 10.1016/j.addr.2013.11.009 24270007 PMC4219254

[B12] Bikram M. West J. L. (2008). Thermo-responsive systems for controlled drug delivery. *Expert Opin. Drug Deliv.* 5 1077–1091. 10.1517/17425247.5.10.1077 18817514

[B13] Bobo D. Robinson K. J. Islam J. Thurecht K. J. Corrie S. R. (2016). Nanoparticle-based medicines: a review of FDA-approved materials and clinical trials to date. *Pharm. Res.* 33 2373–2387. 10.1007/s11095-016-1958-5 27299311

[B14] Boltnarova B. Kubackova J. Skoda J. Stefela A. Smekalova M. Svacinova P. (2021). PLGA based nanospheres as a potent macrophage-specific drug delivery system. *Nanomaterials* 11:749. 10.3390/nano11030749 33809764 PMC8002218

[B15] Brown J. M. Wilson W. R. (2004). Exploiting tumour hypoxia in cancer treatment. *Nat. Rev. Cancer* 4 437–447. 10.1038/nrc1367 15170446

[B16] Butowska K. Woziwodzka A. Borowik A. Piosik J. (2021). Polymeric nanocarriers: a transformation in doxorubicin therapies. *Materials* 14:2135. 10.3390/ma14092135 33922291 PMC8122860

[B17] Cabral H. Kataoka K. (2014). Progress of drug-loaded polymeric micelles into clinical studies. *J. Control Release* 190 465–476. 10.1016/j.jconrel.2014.06.042 24993430

[B18] Cabral H. Miyata K. Kishimura A. (2014). Nanodevices for studying nano-pathophysiology. *Adv. Drug Deliv. Rev.* 74 35–52. 10.1016/j.addr.2014.06.003 24993612

[B19] Caldorera-Moore M. E. Liechty W. B. Peppas N. A. (2011). Responsive theranostic systems: integration of diagnostic imaging agents and responsive controlled release drug delivery carriers. *Acc. Chem. Res.* 44 1061–1070. 10.1021/ar2001777 21932809 PMC3219056

[B20] Callmann C. E. Barback C. V. Thompson M. P. Hall D. J. Mattrey R. F. Gianneschi N. C. (2015). Therapeutic enzyme-responsive nanoparticles for targeted delivery and accumulation in tumors. *Adv. Mater.* 27 4611–4615. 10.1002/adma.201501803 26178920 PMC4699560

[B21] Cao J. Chen D. Huang S. Deng D. Tang L. Gu Y. (2016). Multifunctional near-infrared light-triggered biodegradable micelles for chemo- and photo-thermal combination therapy. *Oncotarget* 7 82170–82184. 10.18632/oncotarget.10320 27366951 PMC5347683

[B22] Cao J. Huang S. Chen Y. Li S. Li X. Deng D. (2013). Near-infrared light-triggered micelles for fast controlled drug release in deep tissue. *Biomaterials* 34 6272–6283. 10.1016/j.biomaterials.2013.05.008 23721796

[B23] Carita A. C. Eloy J. O. Chorilli M. Lee R. J. Leonardi G. R. (2018). Recent advances and perspectives in liposomes for cutaneous drug delivery. *Curr. Med. Chem.* 25 606–635. 10.2174/0929867324666171009120154 28990515

[B24] Caster J. M. Patel A. N. Zhang T. Wang A. (2017). Investigational nanomedicines in 2016: a review of nanotherapeutics currently undergoing clinical trials. *Wiley Interdiscip. Rev. Nanomed. Nanobiotechnol.* 9:e1416. 10.1002/wnan.1416 27312983

[B25] Chandrawati R. (2016). Enzyme-responsive polymer hydrogels for therapeutic delivery. *Exp. Biol. Med.* 241 972–979. 10.1177/1535370216647186 27188515 PMC4950356

[B26] Chang G. Li C. Lu W. Ding J. (2010). N-Boc-histidine-capped PLGA-PEG-PLGA as a smart polymer for drug delivery sensitive to tumor extracellular pH. *Macromol. Biosci.* 10 1248–1256. 10.1002/mabi.201000117 20593367

[B27] Chen Q. Feng L. Liu J. Zhu W. Dong Z. Wu Y. (2016). Intelligent albumin-MnO2 nanoparticles as pH-/H2 O2 -responsive dissociable nanocarriers to modulate tumor hypoxia for effective combination therapy. *Adv. Mater.* 28 7129–7136. 10.1002/adma.201601902 27283434

[B28] Chen Q. Ke H. Dai Z. Liu Z. (2015). Nanoscale theranostics for physical stimulus-responsive cancer therapies. *Biomaterials* 73 214–230. 10.1016/j.biomaterials.2015.09.018 26410788

[B29] Cheng R. Meng F. Deng C. Klok H. A. Zhong Z. (2013). Dual and multi-stimuli responsive polymeric nanoparticles for programmed site-specific drug delivery. *Biomaterials* 34 3647–3657. 10.1016/j.biomaterials.2013.01.084 23415642

[B30] Chiang Y. T. Yen Y. W. Lo C. L. (2015). Reactive oxygen species and glutathione dual redox-responsive micelles for selective cytotoxicity of cancer. *Biomaterials* 61 150–161. 10.1016/j.biomaterials.2015.05.007 26002788

[B31] Chien M. P. Carlini A. S. Hu D. Barback C. V. Rush A. M. Hall D. J. (2013). Enzyme-directed assembly of nanoparticles in tumors monitored by in vivo whole animal imaging and ex vivo super-resolution fluorescence imaging. *J. Am. Chem. Soc.* 135 18710–18713. 10.1021/ja408182p 24308273 PMC4021865

[B32] Choi K. Y. Han H. S. Lee E. S. Shin J. M. Almquist B. D. Lee D. S. (2019). Hyaluronic acid-based activatable nanomaterials for stimuli-responsive imaging and therapeutics: beyond CD44-mediated drug delivery. *Adv. Mater.* 31:e1803549. 10.1002/adma.201803549 30773699

[B33] Cui D. Huang J. Zhen X. Li J. Jiang Y. Pu K. (2019). A semiconducting polymer nano-prodrug for hypoxia-activated photodynamic cancer therapy. *Angew. Chem. Int. Ed. Engl.* 58 5920–5924. 10.1002/anie.201814730 30793456

[B34] Dai Y. Xu C. Sun X. Chen X. (2017). Nanoparticle design strategies for enhanced anticancer therapy by exploiting the tumour microenvironment. *Chem. Soc. Rev.* 46 3830–3852. 10.1039/c6cs00592f 28516983 PMC5521825

[B35] De Jong W. H. Borm P. J. (2008). Drug delivery and nanoparticles:applications and hazards. *Int. J. Nanomed.* 3 133–149. 10.2147/ijn.s596 18686775 PMC2527668

[B36] De Volder M. F. Tawfick S. H. Baughman R. H. Hart A. J. (2013). Carbon nanotubes: present and future commercial applications. *Science* 339 535–539. 10.1126/science.1222453 23372006

[B37] Du J. Lane L. A. Nie S. (2015). Stimuli-responsive nanoparticles for targeting the tumor microenvironment. *J. Control Release* 219 205–214. 10.1016/j.jconrel.2015.08.050 26341694 PMC4656063

[B38] Duncan R. (2003). The dawning era of polymer therapeutics. *Nat. Rev. Drug Discov.* 2 347–360. 10.1038/nrd1088 12750738

[B39] Duncan R. (2006). Polymer conjugates as anticancer nanomedicines. *Nat. Rev. Cancer* 6 688–701. 10.1038/nrc1958 16900224

[B40] Duncan R. Vicent M. J. Greco F. Nicholson R. I. (2005). Polymer-drug conjugates: towards a novel approach for the treatment of endrocine-related cancer. *Endocr. Relat. Cancer* 12(Suppl. 1), S189–S199. 10.1677/erc.1.01045 16113096

[B41] Ekladious I. Colson Y. L. Grinstaff M. W. (2019). Polymer-drug conjugate therapeutics: advances, insights and prospects. *Nat. Rev. Drug Discov.* 18 273–294. 10.1038/s41573-018-0005-0 30542076 PMC12032968

[B42] Fan W. Bu W. Zhang Z. Shen B. Zhang H. He Q. (2015). X-ray radiation-controlled no-release for on-demand depth-independent hypoxic radiosensitization. *Angew. Chem. Int. Ed. Engl.* 54 14026–14030. 10.1002/anie.201504536 26228648

[B43] Fleige E. Quadir M. A. Haag R. (2012). Stimuli-responsive polymeric nanocarriers for the controlled transport of active compounds: concepts and applications. *Adv. Drug Deliv. Rev.* 64 866–884. 10.1016/j.addr.2012.01.020 22349241

[B44] Fu X. Hosta-Rigau L. Chandrawati R. Cui J. (2018). Multi-stimuli-responsive polymer particles, films, and hydrogels for drug delivery. *Chem* 4 2084–2107. 10.1016/j.chempr.2018.07.002

[B45] Gallo J. Kamaly N. Lavdas I. Stevens E. Nguyen Q. D. Wylezinska-Arridge M. (2014). CXCR4-targeted and MMP-responsive iron oxide nanoparticles for enhanced magnetic resonance imaging. *Angew. Chem. Int. Ed. Engl.* 53 9550–9554. 10.1002/anie.201405442 25045009 PMC4321346

[B46] Ganguly S. Dewanjee S. Sen R. Chattopadhyay D. Ganguly S. Gaonkar R. (2021). Apigenin-loaded galactose tailored PLGA nanoparticles: a possible strategy for liver targeting to treat hepatocellular carcinoma. *Colloids Surf. B Biointerfaces* 204:111778. 10.1016/j.colsurfb.2021.111778 33915380

[B47] Ge J. Neofytou E. Cahill T. J. III Beygui R. E. Zare R. N. (2012). Drug release from electric-field-responsive nanoparticles. *ACS Nano* 6 227–233. 10.1021/nn203430m 22111891 PMC3489921

[B48] Grange C. Geninatti-Crich S. Esposito G. Alberti D. Tei L. Bussolati B. (2010). Combined delivery and magnetic resonance imaging of neural cell adhesion molecule-targeted doxorubicin-containing liposomes in experimentally induced Kaposi’s sarcoma. *Cancer Res.* 70 2180–2190. 10.1158/0008-5472.CAN-09-2821 20215497

[B49] Greco F. Vicent M. J. (2009). Combination therapy: opportunities and challenges for polymer-drug conjugates as anticancer nanomedicines. *Adv. Drug Deliv. Rev.* 61 1203–1213. 10.1016/j.addr.2009.05.006 19699247

[B50] Guo H. Zheng Y. Wang B. Li Z. (2015). A note on an improved self-healing group key distribution scheme. *Sensors* 15 25033–25038. 10.3390/s151025033 26426018 PMC4634393

[B51] Hamner K. L. Alexander C. M. Coopersmith K. Reishofer D. Provenza C. Maye M. M. (2013). Using temperature-sensitive smart polymers to regulate DNA-mediated nanoassembly and encoded nanocarrier drug release. *ACS Nano* 7 7011–7020. 10.1021/nn402214e 23899347

[B52] Han H. S. Thambi T. Choi K. Y. Son S. Ko H. Lee M. C. (2015). Bioreducible shell-cross-linked hyaluronic acid nanoparticles for tumor-targeted drug delivery. *Biomacromolecules* 16 447–456. 10.1021/bm5017755 25565417

[B53] Han L. Zhang X. Y. Wang Y. L. Li X. Yang X. H. Huang M. (2017). Redox-responsive theranostic nanoplatforms based on inorganic nanomaterials. *J. Control Release* 259 40–52. 10.1016/j.jconrel.2017.03.018 28288893

[B54] Han L. Zhao J. Zhang X. Cao W. Hu X. Zou G. (2012). Enhanced siRNA delivery and silencing gold-chitosan nanosystem with surface charge-reversal polymer assembly and good biocompatibility. *ACS Nano* 6 7340–7351. 10.1021/nn3024688 22838646

[B55] Hao Y. Zheng C. Wang L. Zhang J. Niu X. Song Q. (2017). Tumor acidity-activatable manganese phosphate nanoplatform for amplification of photodynamic cancer therapy and magnetic resonance imaging. *Acta Biomater.* 62 293–305. 10.1016/j.actbio.2017.08.028 28842332

[B56] Havel H. A. (2016). Where are the nanodrugs? An industry perspective on development of drug products containing nanomaterials. *AAPS J.* 18 1351–1353. 10.1208/s12248-016-9970-6 27520380

[B57] He H. Sun L. Ye J. Liu E. Chen S. Liang Q. (2016). Enzyme-triggered, cell penetrating peptide-mediated delivery of anti-tumor agents. *J. Control Release* 240 67–76. 10.1016/j.jconrel.2015.10.040 26514292

[B58] He H. Zhu R. Sun W. Cai K. Chen Y. Yin L. (2018). Selective cancer treatment via photodynamic sensitization of hypoxia-responsive drug delivery. *Nanoscale* 10 2856–2865. 10.1039/c7nr07677k 29364314

[B59] He J. Li C. Ding L. Huang Y. Yin X. Zhang J. (2019). Tumor targeting strategies of smart fluorescent nanoparticles and their applications in cancer diagnosis and treatment. *Adv. Mater.* 31:e1902409. 10.1002/adma.201902409 31369176

[B60] He T. Jiang C. He J. Zhang Y. He G. Wu J. (2021). Manganese-dioxide-coating-instructed plasmonic modulation of gold nanorods for activatable duplex-imaging-guided NIR-II photothermal-chemodynamic therapy. *Adv. Mater.* 33:e2008540. 10.1002/adma.202008540 33645863

[B61] Her S. Jaffray D. A. Allen C. (2017). Gold nanoparticles for applications in cancer radiotherapy: mechanisms and recent advancements. *Adv. Drug Deliv. Rev.* 109 84–101. 10.1016/j.addr.2015.12.012 26712711

[B62] Hu F. Q. Zhang Y. Y. You J. Yuan H. Du Y. Z. (2012). pH triggered doxorubicin delivery of PEGylated glycolipid conjugate micelles for tumor targeting therapy. *Mol. Pharm* 9 2469–2478. 10.1021/mp300002v 22827551

[B63] Hu Q. Katti P. S. Gu Z. (2014). Enzyme-responsive nanomaterials for controlled drug delivery. *Nanoscale* 6 12273–12286. 10.1039/c4nr04249b 25251024 PMC4425417

[B64] Hua S. de Matos M. B. C. Metselaar J. M. Storm G. (2018). Current trends and challenges in the clinical translation of nanoparticulate nanomedicines: pathways for translational development and commercialization. *Front. Pharmacol.* 9:790. 10.3389/fphar.2018.00790 30065653 PMC6056679

[B65] Huang D. Sun L. Huang L. Chen Y. (2021). Nanodrug delivery systems modulate tumor vessels to increase the enhanced permeability and retention effect. *J. Pers. Med.* 11:124. 10.3390/jpm11020124 33672813 PMC7917988

[B66] Jackson E. F. Esparza-Coss E. Wen X. Ng C. S. Daniel S. L. Price R. E. (2007). Magnetic resonance imaging of therapy-induced necrosis using gadolinium-chelated polyglutamic acids. *Int. J. Radiat. Oncol. Biol. Phys.* 68 830–838. 10.1016/j.ijrobp.2007.01.011 17379450 PMC1997292

[B67] Jaidev L. R. Chellappan D. R. Bhavsar D. V. Ranganathan R. Sivanantham B. Subramanian A. (2017). Multi-functional nanoparticles as theranostic agents for the treatment & imaging of pancreatic cancer. *Acta Biomater.* 49 422–433. 10.1016/j.actbio.2016.11.053 27890622

[B68] Jain R. K. Stylianopoulos T. (2010). Delivering nanomedicine to solid tumors. *Nat. Rev. Clin. Oncol.* 7 653–664. 10.1038/nrclinonc.2010.139 20838415 PMC3065247

[B69] Jia L. Li Z. Zhang D. Zhang Q. Shen J. Guo H. (2013). Redox-responsive catiomer based on PEG-ss-chitosan oligosaccharide-ss-polyethylenimine copolymer for effective gene delivery. *Polym. Chem.* 4 156–165. 10.1039/C2PY20781H

[B70] Jiang M. Mu J. Jacobson O. Wang Z. He L. Zhang F. (2020). Reactive oxygen species activatable heterodimeric prodrug as tumor-selective nanotheranostics. *ACS Nano* 14 16875–16886. 10.1021/acsnano.0c05722 33206522

[B71] Jiang W. Zhou Y. Yan D. (2015). Hyperbranched polymer vesicles: from self-assembly, characterization, mechanisms, and properties to applications. *Chem. Soc. Rev.* 44 3874–3889. 10.1039/c4cs00274a 25336064

[B72] Jin Z. Wen Y. Hu Y. Chen W. Zheng X. Guo W. (2017). MRI-guided and ultrasound-triggered release of NO by advanced nanomedicine. *Nanoscale* 9 3637–3645. 10.1039/c7nr00231a 28247895

[B73] Johannsen M. Gneveckow U. Eckelt L. Feussner A. Waldofner N. Scholz R. (2005). Clinical hyperthermia of prostate cancer using magnetic nanoparticles: presentation of a new interstitial technique. *Int. J. Hypertherm.* 21 637–647. 10.1080/02656730500158360 16304715

[B74] Kakwere H. Leal M. P. Materia M. E. Curcio A. Guardia P. Niculaes D. (2015). Functionalization of strongly interacting magnetic nanocubes with (thermo)responsive coating and their application in hyperthermia and heat-triggered drug delivery. *ACS Appl. Mater. Interfaces* 7 10132–10145. 10.1021/am5088117 25840122

[B75] Kamaly N. Yameen B. Wu J. Farokhzad O. C. (2016). Degradable controlled-release polymers and polymeric nanoparticles: mechanisms of controlling drug release. *Chem. Rev.* 116 2602–2663. 10.1021/acs.chemrev.5b00346 26854975 PMC5509216

[B76] Kanamala M. Wilson W. R. Yang M. Palmer B. D. Wu Z. (2016). Mechanisms and biomaterials in pH-responsive tumour targeted drug delivery: a review. *Biomaterials* 85 152–167. 10.1016/j.biomaterials.2016.01.061 26871891

[B77] Karimi M. Ghasemi A. Sahandi Zangabad P. Rahighi R. Moosavi Basri S. M. Mirshekari H. (2016). Smart micro/nanoparticles in stimulus-responsive drug/gene delivery systems. *Chem. Soc. Rev.* 45 1457–1501. 10.1039/c5cs00798d 26776487 PMC4775468

[B78] Ke W. Li J. Mohammed F. Wang Y. Tou K. Liu X. (2019). Therapeutic polymersome nanoreactors with tumor-specific activable cascade reactions for cooperative cancer therapy. *ACS Nano* 13 2357–2369. 10.1021/acsnano.8b09082 30699292

[B79] Khokha R. Murthy A. Weiss A. (2013). Metalloproteinases and their natural inhibitors in inflammation and immunity. *Nat. Rev. Immunol.* 13 649–665. 10.1038/nri3499 23969736

[B80] Kim J. Kim J. Jeong C. Kim W. J. (2016). Synergistic nanomedicine by combined gene and photothermal therapy. *Adv. Drug Deliv. Rev.* 98 99–112. 10.1016/j.addr.2015.12.018 26748259

[B81] Kolosnjaj-Tabi J. Gibot L. Fourquaux I. Golzio M. Rols M. P. (2019). Electric field-responsive nanoparticles and electric fields: physical, chemical, biological mechanisms and therapeutic prospects. *Adv. Drug Deliv. Rev.* 138 56–67. 10.1016/j.addr.2018.10.017 30414494

[B82] Kulkarni A. Rao P. Natarajan S. Goldman A. Sabbisetti V. S. Khater Y. (2016). Reporter nanoparticle that monitors its anticancer efficacy in real time. *Proc. Natl. Acad. Sci. U.S.A.* 113 E2104–E2113. 10.1073/pnas.1603455113 27036008 PMC4839457

[B83] Kulkarni P. Haldar M. K. You S. Choi Y. Mallik S. (2016). Hypoxia-responsive polymersomes for drug delivery to hypoxic pancreatic cancer cells. *Biomacromolecules* 17 2507–2513. 10.1021/acs.biomac.6b00350 27303825 PMC5502721

[B84] Kumar S. Rani R. Dilbaghi N. Tankeshwar K. Kim K. H. (2017). Carbon nanotubes: a novel material for multifaceted applications in human healthcare. *Chem. Soc. Rev.* 46 158–196. 10.1039/c6cs00517a 27841412

[B85] Lai J. Shah B. P. Zhang Y. Yang L. Lee K. B. (2015). Real-time monitoring of ATP-responsive drug release using mesoporous-silica-coated multicolor upconversion nanoparticles. *ACS Nano* 9 5234–5245. 10.1021/acsnano.5b00641 25859611 PMC5808884

[B86] Le Fevre R. Durand-Dubief M. Chebbi I. Mandawala C. Lagroix F. Valet J. P. (2017). Enhanced antitumor efficacy of biocompatible magnetosomes for the magnetic hyperthermia treatment of glioblastoma. *Theranostics* 7 4618–4631. 10.7150/thno.18927 29158849 PMC5695153

[B87] Lee K. S. Chung H. C. Im S. A. Park Y. H. Kim C. S. Kim S. B. (2008). Multicenter phase II trial of Genexol-PM, a Cremophor-free, polymeric micelle formulation of paclitaxel, in patients with metastatic breast cancer. *Breast Cancer Res. Treat* 108 241–250. 10.1007/s10549-007-9591-y 17476588

[B88] Lee K. Bae K. H. Lee Y. Lee S. H. Ahn C. H. Park T. G. (2010). Pluronic/polyethylenimine shell crosslinked nanocapsules with embedded magnetite nanocrystals for magnetically triggered delivery of siRNA. *Macromol. Biosci.* 10 239–245. 10.1002/mabi.200900291 19924685

[B89] Li D. Ma Y. Du J. Tao W. Du X. Yang X. (2017a). Tumor acidity/NIR controlled interaction of transformable nanoparticle with biological systems for cancer therapy. *Nano Lett.* 17 2871–2878. 10.1021/acs.nanolett.6b05396 28375632

[B90] Li J. Pu K. (2020). Semiconducting polymer nanomaterials as near-infrared photoactivatable protherapeutics for cancer. *Acc. Chem. Res.* 53 752–762. 10.1021/acs.accounts.9b00569 32027481

[B91] Li J. Chen Q. Zha Z. Li H. Toh K. Dirisala A. (2015). Ternary polyplex micelles with PEG shells and intermediate barrier to complexed DNA cores for efficient systemic gene delivery. *J. Control Release* 209 77–87. 10.1016/j.jconrel.2015.04.024 25912408

[B92] Li J. Dirisala A. Ge Z. Wang Y. Yin W. Ke W. (2017b). Therapeutic vesicular nanoreactors with tumor-specific activation and self-destruction for synergistic tumor ablation. *Angew. Chem. Int. Ed. Engl.* 56 14025–14030. 10.1002/anie.201706964 28940903

[B93] Li J. Li Y. Wang Y. Ke W. Chen W. Wang W. (2017c). Polymer prodrug-based nanoreactors activated by tumor acidity for orchestrated oxidation/chemotherapy. *Nano Lett.* 17 6983–6990. 10.1021/acs.nanolett.7b03531 28977746

[B94] Li J. Liu F. Shao Q. Min Y. Costa M. Yeow E. K. (2014). Enzyme-responsive cell-penetrating peptide conjugated mesoporous silica quantum dot nanocarriers for controlled release of nucleus-targeted drug molecules and real-time intracellular fluorescence imaging of tumor cells. *Adv. Healthc. Mater.* 3 1230–1239. 10.1002/adhm.201300613 24550203

[B95] Li K. Liu B. (2014). Polymer-encapsulated organic nanoparticles for fluorescence and photoacoustic imaging. *Chem. Soc. Rev.* 43 6570–6597. 10.1039/c4cs00014e 24792930

[B96] Li Y. Yang H. Y. Lee D. S. (2016). Polymer-based and pH-sensitive nanobiosensors for imaging and therapy of acidic pathological areas. *Pharm. Res.* 33 2358–2372. 10.1007/s11095-016-1944-y 27183840

[B97] Ling D. Park W. Park S. J. Lu Y. Kim K. S. Hackett M. J. (2014). Multifunctional tumor pH-sensitive self-assembled nanoparticles for bimodal imaging and treatment of resistant heterogeneous tumors. *J. Am. Chem. Soc.* 136 5647–5655. 10.1021/ja4108287 24689550

[B98] Liu F. Lou J. Hristov D. (2017). X-Ray responsive nanoparticles with triggered release of nitrite, a precursor of reactive nitrogen species, for enhanced cancer radiosensitization. *Nanoscale* 9 14627–14634. 10.1039/c7nr04684g 28936509

[B99] Liu J. F. Neel N. Dang P. Lamb M. McKenna J. Rodgers L. (2018). Radiofrequency-triggered drug release from nanoliposomes with millimeter-scale resolution using a superimposed static gating field. *Small* 14:e1802563. 10.1002/smll.201802563 30286280 PMC6397654

[B100] Liu J. Huang Y. Kumar A. Tan A. Jin S. Mozhi A. (2014). pH-sensitive nano-systems for drug delivery in cancer therapy. *Biotechnol. Adv.* 32 693–710. 10.1016/j.biotechadv.2013.11.009 24309541

[B101] Liu P. Shi B. Yue C. Gao G. Li P. Yi H. (2013). Dextran-based redox-responsive doxorubicin prodrug micelles for overcoming multidrug resistance. *Polym. Chem.* 4 5793–5799. 10.1039/C3PY00830D

[B102] Liu T. Wang C. Gu X. Gong H. Cheng L. Shi X. (2014). Drug delivery with PEGylated MoS2 nano-sheets for combined photothermal and chemotherapy of cancer. *Adv. Mater.* 26 3433–3440. 10.1002/adma.201305256 24677423

[B103] Liu Y. Bhattarai P. Dai Z. Chen X. (2019). Photothermal therapy and photoacoustic imaging via nanotheranostics in fighting cancer. *Chem. Soc. Rev.* 48 2053–2108. 10.1039/c8cs00618k 30259015 PMC6437026

[B104] Lucky S. S. Soo K. C. Zhang Y. (2015). Nanoparticles in photodynamic therapy. *Chem. Rev.* 115 1990–2042. 10.1021/cr5004198 25602130

[B105] Luk B. T. Zhang L. (2014). Current advances in polymer-based nanotheranostics for cancer treatment and diagnosis. *ACS Appl. Mater. Interfaces* 6 21859–21873. 10.1021/am5036225 25014486 PMC4278687

[B106] Luk B. T. Fang R. H. Zhang L. (2012). Lipid- and polymer-based nanostructures for cancer theranostics. *Theranostics* 2 1117–1126. 10.7150/thno.4381 23382770 PMC3563151

[B107] Luque-Michel E. Imbuluzqueta E. Sebastian V. Blanco-Prieto M. J. (2017). Clinical advances of nanocarrier-based cancer therapy and diagnostics. *Expert Opin. Drug Deliv.* 14 75–92. 10.1080/17425247.2016.1205585 27339650

[B108] Ma Y. Mou Q. Wang D. Zhu X. Yan D. (2016). Dendritic polymers for theranostics. *Theranostics* 6 930–947. 10.7150/thno.14855 27217829 PMC4876620

[B109] Madaan K. Kumar S. Poonia N. Lather V. Pandita D. (2014). Dendrimers in drug delivery and targeting: drug-dendrimer interactions and toxicity issues. *J. Pharm. Bioallied Sci.* 6 139–150. 10.4103/0975-7406.130965 25035633 PMC4097927

[B110] Maeda H. (2021). The 35th anniversary of the discovery of EPR effect: a new wave of nanomedicines for tumor-targeted drug delivery-personal remarks and future prospects. *J. Pers. Med.* 11:229. 10.3390/jpm11030229 33810037 PMC8004895

[B111] Maeda H. Bharate G. Y. Daruwalla J. (2009). Polymeric drugs for efficient tumor-targeted drug delivery based on EPR-effect. *Eur. J. Pharm. Biopharm.* 71 409–419. 10.1016/j.ejpb.2008.11.010 19070661

[B112] Mai B. T. Balakrishnan P. B. Barthel M. J. Piccardi F. Niculaes D. Marinaro F. (2019). Thermoresponsive iron oxide nanocubes for an effective clinical translation of magnetic hyperthermia and heat-mediated chemotherapy. *ACS Appl. Mater. Interfaces* 11 5727–5739. 10.1021/acsami.8b16226 30624889 PMC6376448

[B113] Majumder J. Minko T. (2021). Multifunctional and stimuli-responsive nanocarriers for targeted therapeutic delivery. *Expert Opin. Drug Deliv.* 18 205–227. 10.1080/17425247.2021.1828339 32969740 PMC7904578

[B114] Martinelli C. Pucci C. Ciofani G. (2019). Nanostructured carriers as innovative tools for cancer diagnosis and therapy. *APL Bioeng.* 3:011502. 10.1063/1.5079943 PMC6481740 31069332

[B115] McHugh K. J. Jing L. Behrens A. M. Jayawardena S. Tang W. Gao M. (2018). Biocompatible semiconductor quantum dots as cancer imaging agents. *Adv. Mater.* 30:e1706356. 10.1002/adma.201706356 29468747

[B116] Mi P. Cabral H. Kataoka K. (2020). Ligand-installed nanocarriers toward precision therapy. *Adv. Mater.* 32:e1902604. 10.1002/adma.201902604 31353770

[B117] Mitchell M. J. Billingsley M. M. Haley R. M. Wechsler M. E. Peppas N. A. Langer R. (2021). Engineering precision nanoparticles for drug delivery. *Nat. Rev. Drug Discov.* 20 101–124. 10.1038/s41573-020-0090-8 33277608 PMC7717100

[B118] Mohamed S. M. Veeranarayanan S. Maekawa T. Kumar S. (2019). External stimulus responsive inorganic nanomaterials for cancer theranostics. *Adv. Drug Deliv. Rev.* 138 18–40. 10.1016/j.addr.2018.10.007 30321621

[B119] Montero de Espinosa L. Meesorn W. Moatsou D. Weder C. (2017). Bioinspired polymer systems with stimuli-responsive mechanical properties. *Chem. Rev.* 117 12851–12892. 10.1021/acs.chemrev.7b00168 28752995

[B120] Mu J. Lin J. Huang P. Chen X. (2018). Development of endogenous enzyme-responsive nanomaterials for theranostics. *Chem. Soc. Rev.* 47 5554–5573. 10.1039/c7cs00663b 29856446 PMC6066418

[B121] Neradovic D. Soga O. Van Nostrum C. F. Hennink W. E. (2004). The effect of the processing and formulation parameters on the size of nanoparticles based on block copolymers of poly(ethylene glycol) and poly(N-isopropylacrylamide) with and without hydrolytically sensitive groups. *Biomaterials* 25 2409–2418. 10.1016/j.biomaterials.2003.09.024 14741606

[B122] Nguyen Q. T. Tsien R. Y. (2013). Fluorescence-guided surgery with live molecular navigation–a new cutting edge. *Nat. Rev. Cancer* 13 653–662. 10.1038/nrc3566 23924645 PMC4427343

[B123] Oerlemans C. Bult W. Bos M. Storm G. Nijsen J. F. Hennink W. E. (2010). Polymeric micelles in anticancer therapy: targeting, imaging and triggered release. *Pharm. Res.* 27 2569–2589. 10.1007/s11095-010-0233-4 20725771 PMC2982955

[B124] Overchuk M. Zheng G. (2018). Overcoming obstacles in the tumor microenvironment: recent advancements in nanoparticle delivery for cancer theranostics. *Biomaterials* 156 217–237. 10.1016/j.biomaterials.2017.10.024 29207323

[B125] Paris J. L. Cabanas M. V. Manzano M. Vallet-Regi M. (2015). Polymer-grafted mesoporous silica nanoparticles as ultrasound-responsive drug carriers. *ACS Nano* 9 11023–11033. 10.1021/acsnano.5b04378 26456489

[B126] Perche F. Biswas S. Wang T. Zhu L. Torchilin V. P. (2014). Hypoxia-targeted siRNA delivery. *Angew. Chem. Int. Ed. Engl.* 53 3362–3366. 10.1002/anie.201308368 24554550 PMC4150469

[B127] Pham S. H. Choi Y. Choi J. (2020). Stimuli-responsive nanomaterials for application in antitumor therapy and drug delivery. *Pharmaceutics* 12:630. 10.3390/pharmaceutics12070630 32635539 PMC7408499

[B128] Prabhu P. Patravale V. (2012). The upcoming field of theranostic nanomedicine: an overview. *J. Biomed. Nanotechnol.* 8 859–882. 10.1166/jbn.2012.1459 23029995

[B129] Prasad P. N. (2012). *Introduction to Nanomedicine and Nanobioengineering.* Hoboken, NJ: John Wiley & Sons.

[B130] Qi C. He J. Fu L. H. He T. Blum N. T. Yao X. (2021). Tumor-specific activatable nanocarriers with gas-generation and signal amplification capabilities for tumor theranostics. *ACS Nano* 15 1627–1639. 10.1021/acsnano.0c09223 33356128

[B131] Qin S. Geng Y. Discher D. E. Yang S. (2006). Temperature-controlled assembly and release from polymer vesicles of Poly(ethylene oxide)-block- poly(N-isopropylacrylamide). *Adv. Mater.* 18 2905–2909. 10.1002/adma.200601019

[B132] Ramos J. Forcada J. Hidalgo-Alvarez R. (2014). Cationic polymer nanoparticles and nanogels: from synthesis to biotechnological applications. *Chem. Rev.* 114 367–428. 10.1021/cr3002643 24003911

[B133] Rampersaud S. Fang J. Wei Z. Fabijanic K. Silver S. Jaikaran T. (2016). The effect of cage shape on nanoparticle-based drug carriers: anticancer drug release and efficacy via receptor blockade using dextran-coated iron oxide nanocages. *Nano Lett.* 16 7357–7363. 10.1021/acs.nanolett.6b02577 27960523 PMC5610656

[B134] Rao N. V. Ko H. Lee J. Park J. H. (2018). Recent progress and advances in stimuli-responsive polymers for cancer therapy. *Front. Bioeng. Biotechnol.* 6:110. 10.3389/fbioe.2018.00110 30159310 PMC6104418

[B135] Rejinold N. S. Jayakumar R. Kim Y. C. (2015). Radio frequency responsive nano-biomaterials for cancer therapy. *J. Control Release* 204 85–97. 10.1016/j.jconrel.2015.02.036 25744825

[B136] Ren J. M. McKenzie T. G. Fu Q. Wong E. H. Xu J. An Z. (2016). Star polymers. *Chem. Rev.* 116 6743–6836. 10.1021/acs.chemrev.6b00008 27299693

[B137] Rizzo L. Y. Theek B. Storm G. Kiessling F. Lammers T. (2013). Recent progress in nanomedicine: therapeutic, diagnostic and theranostic applications. *Curr. Opin. Biotechnol.* 24 1159–1166. 10.1016/j.copbio.2013.02.020 23578464 PMC3833836

[B138] Rosenblum D. Joshi N. Tao W. Karp J. M. Peer D. (2018). Progress and challenges towards targeted delivery of cancer therapeutics. *Nat. Commun.* 9:1410. 10.1038/s41467-018-03705-y 29650952 PMC5897557

[B139] Sadhukha T. Wiedmann T. S. Panyam J. (2013). Inhalable magnetic nanoparticles for targeted hyperthermia in lung cancer therapy. *Biomaterials* 34 5163–5171. 10.1016/j.biomaterials.2013.03.061 23591395 PMC4673896

[B140] Sahay G. Alakhova D. Y. Kabanov A. V. (2010). Endocytosis of nanomedicines. *J. Control Release* 145 182–195. 10.1016/j.jconrel.2010.01.036 20226220 PMC2902597

[B141] Saravanakumar G. Kim J. Kim W. J. (2017). Reactive-oxygen-species-responsive drug delivery systems: promises and challenges. *Adv. Sci.* 4:1600124. 10.1002/advs.201600124 28105390 PMC5238745

[B142] Schadlich A. Caysa H. Mueller T. Tenambergen F. Rose C. Gopferich A. (2011). Tumor accumulation of NIR fluorescent PEG-PLA nanoparticles: impact of particle size and human xenograft tumor model. *ACS Nano* 5 8710–8720. 10.1021/nn2026353 21970766

[B143] Schmidt M. M. Wittrup K. D. (2009). A modeling analysis of the effects of molecular size and binding affinity on tumor targeting. *Mol. Cancer Ther.* 8 2861–2871. 10.1158/1535-7163.MCT-09-0195 19825804 PMC4078872

[B144] Senapati S. Mahanta A. K. Kumar S. Maiti P. (2018). Controlled drug delivery vehicles for cancer treatment and their performance. *Signal. Transduct. Target Ther.* 3:7. 10.1038/s41392-017-0004-3 29560283 PMC5854578

[B145] Sharma H. Mishra P. K. Talegaonkar S. Vaidya B. (2015). Metal nanoparticles: a theranostic nanotool against cancer. *Drug Discov. Today* 20 1143–1151. 10.1016/j.drudis.2015.05.009 26007605

[B146] Sharma R. Mody N. Agrawal U. Vyas S. P. (2017). Theranostic nanomedicine; a next generation platform for cancer diagnosis and therapy. *Mini Rev. Med. Chem.* 17 1746–1757. 10.2174/1389557516666160219122524 26891932

[B147] Shi J. Kantoff P. W. Wooster R. Farokhzad O. C. (2017). Cancer nanomedicine: progress, challenges and opportunities. *Nat. Rev. Cancer* 17 20–37. 10.1038/nrc.2016.108 27834398 PMC5575742

[B148] Shim G. Ko S. Kim D. Le Q. V. Park G. T. Lee J. (2017). Light-switchable systems for remotely controlled drug delivery. *J. Control Release* 267 67–79. 10.1016/j.jconrel.2017.09.009 28888917

[B149] Shim J. Seok Kang H. Park W. S. Han S. H. Kim J. Chang I. S. (2004). Transdermal delivery of mixnoxidil with block copolymer nanoparticles. *J. Control Release* 97 477–484. 10.1016/j.jconrel.2004.03.028 15212879

[B150] Son J. Yi G. Yoo J. Park C. Koo H. Choi H. S. (2019). Light-responsive nanomedicine for biophotonic imaging and targeted therapy. *Adv. Drug Deliv. Rev.* 138 133–147. 10.1016/j.addr.2018.10.002 30321619 PMC6547138

[B151] Spill F. Reynolds D. S. Kamm R. D. Zaman M. H. (2016). Impact of the physical microenvironment on tumor progression and metastasis. *Curr. Opin. Biotechnol.* 40 41–48. 10.1016/j.copbio.2016.02.007 26938687 PMC4975620

[B152] Sung H. Ferlay J. Siegel R. L. Laversanne M. Soerjomataram I. Jemal A. (2021). Global cancer statistics 2020: GLOBOCAN estimates of incidence and mortality worldwide for 36 cancers in 185 countries. *CA Cancer J. Clin*. 71 209–249. 10.3322/caac.21660 33538338

[B153] Synatschke C. V. Nomoto T. Cabral H. Fortsch M. Toh K. Matsumoto Y. (2014). Multicompartment micelles with adjustable poly(ethylene glycol) shell for efficient in vivo photodynamic therapy. *ACS Nano* 8 1161–1172. 10.1021/nn4028294 24386876

[B154] Tanner P. Baumann P. Enea R. Onaca O. Palivan C. Meier W. (2011). Polymeric vesicles: from drug carriers to nanoreactors and artificial organelles. *Acc. Chem. Res.* 44 1039–1049. 10.1021/ar200036k 21608994

[B155] Thambi T. Deepagan V. G. Yoon H. Y. Han H. S. Kim S. H. Son S. (2014). Hypoxia-responsive polymeric nanoparticles for tumor-targeted drug delivery. *Biomaterials* 35 1735–1743. 10.1016/j.biomaterials.2013.11.022 24290696

[B156] Thambi T. Park J. H. Lee D. S. (2016). Stimuli-responsive polymersomes for cancer therapy. *Biomater. Sci.* 4 55–69. 10.1039/c5bm00268k 26456625

[B157] Uthaman S. Huh K. M. Park I. K. (2018). Tumor microenvironment-responsive nanoparticles for cancer theragnostic applications. *Biomater. Res.* 22:22. 10.1186/s40824-018-0132-z 30155269 PMC6108142

[B158] Vaidya A. Sun Y. Ke T. Jeong E. K. Lu Z. R. (2006). Contrast enhanced MRI-guided photodynamic therapy for site-specific cancer treatment. *Magn. Reson. Med.* 56 761–767. 10.1002/mrm.21009 16902981

[B159] Valle J. W. Armstrong A. Newman C. Alakhov V. Pietrzynski G. Brewer J. (2011). A phase 2 study of SP1049C, doxorubicin in P-glycoprotein-targeting pluronics, in patients with advanced adenocarcinoma of the esophagus and gastroesophageal junction. *Invest. New Drugs* 29 1029–1037. 10.1007/s10637-010-9399-1 20179989

[B160] Vandenbroucke R. E. Libert C. (2014). Is there new hope for therapeutic matrix metalloproteinase inhibition? *Nat. Rev. Drug Discov.* 13 904–927. 10.1038/nrd4390 25376097

[B161] Ventola C. L. (2017). Progress in nanomedicine: approved and investigational nanodrugs. *P T* 42 742–755. 29234213 PMC5720487

[B162] Vicent M. J. Duncan R. (2006). Polymer conjugates: nanosized medicines for treating cancer. *Trends Biotechnol.* 24 39–47. 10.1016/j.tibtech.2005.11.006 16307811

[B163] Vigderman L. Zubarev E. R. (2013). Therapeutic platforms based on gold nanoparticles and their covalent conjugates with drug molecules. *Adv. Drug Deliv. Rev.* 65 663–676. 10.1016/j.addr.2012.05.004 22613038

[B164] Wan Z. Mao H. Guo M. Li Y. Zhu A. Yang H. (2014). Highly efficient hierarchical micelles integrating photothermal therapy and singlet oxygen-synergized chemotherapy for cancer eradication. *Theranostics* 4 399–411. 10.7150/thno.8171 24578723 PMC3936292

[B165] Wang C. Xu L. Liang C. Xiang J. Peng R. Liu Z. (2014). Immunological responses triggered by photothermal therapy with carbon nanotubes in combination with anti-CTLA-4 therapy to inhibit cancer metastasis. *Adv. Mater.* 26 8154–8162. 10.1002/adma.201402996 25331930

[B166] Wang F. Xiao J. Chen S. Sun H. Yang B. Jiang J. (2018). Polymer vesicles: modular platforms for cancer theranostics. *Adv. Mater.* 30:e1705674. 10.1002/adma.201705674 29450915

[B167] Wang G. D. Nguyen H. T. Chen H. Cox P. B. Wang L. Nagata K. (2016a). X-ray induced photodynamic therapy: a combination of radiotherapy and photodynamic therapy. *Theranostics* 6 2295–2305. 10.7150/thno.16141 27877235 PMC5118595

[B168] Wang H. X. Yang X. Z. Sun C. Y. Mao C. Q. Zhu Y. H. Wang J. (2014). Matrix metalloproteinase 2-responsive micelle for siRNA delivery. *Biomaterials* 35 7622–7634. 10.1016/j.biomaterials.2014.05.050 24929619

[B169] Wang J. Yang G. Guo X. Tang Z. Zhong Z. Zhou S. (2014). Redox-responsive polyanhydride micelles for cancer therapy. *Biomaterials* 35 3080–3090. 10.1016/j.biomaterials.2013.12.025 24388799

[B170] Wang R. Yang H. Khan A. R. Yang X. Xu J. Ji J. (2021). Redox-responsive hyaluronic acid-based nanoparticles for targeted photodynamic therapy/chemotherapy against breast cancer. *J. Colloid Interface Sci.* 598 213–228. 10.1016/j.jcis.2021.04.056 33901847

[B171] Wang S. Huang P. Chen X. (2016b). Hierarchical targeting strategy for enhanced tumor tissue accumulation/retention and cellular internalization. *Adv. Mater.* 28 7340–7364. 10.1002/adma.201601498 27255214 PMC5014563

[B172] Wang S. Huang P. Chen X. (2016c). Stimuli-responsive programmed specific targeting in nanomedicine. *ACS Nano* 10 2991–2994. 10.1021/acsnano.6b00870 26881288 PMC5223089

[B173] Wang S. Ma X. Hong X. Cheng Y. Tian Y. Zhao S. (2018). Adjuvant photothermal therapy inhibits local recurrences after breast-conserving surgery with little skin damage. *ACS Nano* 12 662–670. 10.1021/acsnano.7b07757 29271636

[B174] Wang Y. A. Li X. L. Mo Y. Z. Fan C. M. Tang L. Xiong F. (2018). Effects of tumor metabolic microenvironment on regulatory T cells. *Mol. Cancer* 17:168. 10.1186/s12943-018-0913-y 30477520 PMC6260778

[B175] Wang Y. Xie Y. Li J. Peng Z. H. Sheinin Y. Zhou J. (2017). Tumor-penetrating nanoparticles for enhanced anticancer activity of combined photodynamic and hypoxia-activated therapy. *ACS Nano* 11 2227–2238. 10.1021/acsnano.6b08731 28165223 PMC5332348

[B176] Wang Z. Niu G. Chen X. (2014). Polymeric materials for theranostic applications. *Pharm. Res.* 31 1358–1376. 10.1007/s11095-013-1103-7 23765400

[B177] Wei J. Shuai X. Wang R. He X. Li Y. Ding M. (2017). Clickable and imageable multiblock polymer micelles with magnetically guided and PEG-switched targeting and release property for precise tumor theranosis. *Biomaterials* 145 138–153. 10.1016/j.biomaterials.2017.08.005 28863308

[B178] Weiss G. J. Chao J. Neidhart J. D. Ramanathan R. K. Bassett D. Neidhart J. A. (2013). First-in-human phase 1/2a trial of CRLX101, a cyclodextrin-containing polymer-camptothecin nanopharmaceutical in patients with advanced solid tumor malignancies. *Invest. New Drugs* 31 986–1000. 10.1007/s10637-012-9921-8 23397498 PMC3774600

[B179] Wen C. J. Zhang L. W. Al-Suwayeh S. A. Yen T. C. Fang J. Y. (2012). Theranostic liposomes loaded with quantum dots and apomorphine for brain targeting and bioimaging. *Int. J. Nanomed.* 7 1599–1611. 10.2147/IJN.S29369 22619515 PMC3356172

[B180] Wong B. S. Yoong S. L. Jagusiak A. Panczyk T. Ho H. K. Ang W. H. (2013). Carbon nanotubes for delivery of small molecule drugs. *Adv. Drug Deliv. Rev.* 65 1964–2015. 10.1016/j.addr.2013.08.005 23954402

[B181] Wong P. T. Choi S. K. (2015). Mechanisms of drug release in nanotherapeutic delivery systems. *Chem. Rev.* 115 3388–3432. 10.1021/cr5004634 25914945

[B182] Xie A. Hanif S. Ouyang J. Tang Z. Kong N. Kim N. Y. (2020). Stimuli-responsive prodrug-based cancer nanomedicine. *EBioMedicine* 56:102821. 10.1016/j.ebiom.2020.102821 32505922 PMC7280365

[B183] Xie J. Lee S. Chen X. (2010). Nanoparticle-based theranostic agents. *Adv. Drug Deliv. Rev.* 62 1064–1079. 10.1016/j.addr.2010.07.009 20691229 PMC2988080

[B184] Xing H. Hwang K. Lu Y. (2016). Recent developments of liposomes as nanocarriers for theranostic applications. *Theranostics* 6 1336–1352. 10.7150/thno.15464 27375783 PMC4924503

[B185] Xiong X. B. Lavasanifar A. (2011). Traceable multifunctional micellar nanocarriers for cancer-targeted co-delivery of MDR-1 siRNA and doxorubicin. *ACS Nano* 5 5202–5213. 10.1021/nn2013707 21627074

[B186] Xu C. Jiang Y. Han Y. Pu K. Zhang R. (2021). A polymer multicellular nanoengager for synergistic NIR-II photothermal immunotherapy. *Adv. Mater.* 33:e2008061. 10.1002/adma.202008061 33634897

[B187] Yang Y. (2015). Cancer immunotherapy: harnessing the immune system to battle cancer. *J. Clin. Invest.* 125 3335–3337. 10.1172/JCI83871 26325031 PMC4588312

[B188] Yong Y. Cheng X. Bao T. Zu M. Yan L. Yin W. (2015). Tungsten sulfide quantum dots as multifunctional nanotheranostics for in vivo dual-modal image-guided photothermal/radiotherapy synergistic therapy. *ACS Nano* 9 12451–12463. 10.1021/acsnano.5b05825 26495962

[B189] Yousef S. Alsaab H. O. Sau S. Iyer A. K. (2018). Development of asialoglycoprotein receptor directed nanoparticles for selective delivery of curcumin derivative to hepatocellular carcinoma. *Heliyon* 4:e01071. 10.1016/j.heliyon.2018.e01071 30603704 PMC6305692

[B190] Yu J. Chu X. Hou Y. (2014). Stimuli-responsive cancer therapy based on nanoparticles. *Chem. Commun.* 50 11614–11630. 10.1039/c4cc03984j 25058003

[B191] Yu Y. Zhang X. Qiu L. (2014). The anti-tumor efficacy of curcumin when delivered by size/charge-changing multistage polymeric micelles based on amphiphilic poly(beta-amino ester) derivates. *Biomaterials* 35 3467–3479. 10.1016/j.biomaterials.2013.12.096 24439418

[B192] Yu Z. Sun Q. Pan W. Li N. Tang B. (2015). A near-infrared triggered nanophotosensitizer inducing domino effect on mitochondrial reactive oxygen species burst for cancer therapy. *ACS Nano* 9 11064–11074. 10.1021/acsnano.5b04501 26456218

[B193] Yuan Y. Y. Mao C. Q. Du X. J. Du J. Z. Wang F. Wang J. (2012). Surface charge switchable nanoparticles based on zwitterionic polymer for enhanced drug delivery to tumor. *Adv. Mater.* 24 5476–5480. 10.1002/adma.201202296 22886872

[B194] Zhang P. Gao D. An K. Shen Q. Wang C. Zhang Y. (2020). A programmable polymer library that enables the construction of stimuli-responsive nanocarriers containing logic gates. *Nat. Chem.* 12 381–390. 10.1038/s41557-020-0426-3 32152477

[B195] Zhang X. Han L. Liu M. Wang K. Tao L. Wan Q. (2017). Recent progress and advances in redox-responsive polymers as controlled delivery nanoplatforms. *Mater. Chem. Front.* 1 807–822. 10.1039/C6QM00135A

[B196] Zhang Y. Bo S. Feng T. Qin X. Wan Y. Jiang S. (2019). A versatile theranostic nanoemulsion for architecture-dependent multimodal imaging and dually augmented photodynamic therapy. *Adv. Mater.* 31:e1806444. 10.1002/adma.201806444 30907469

[B197] Zhao H. Duong H. H. Yung L. Y. (2010). Folate-conjugated polymer micelles with pH-triggered drug release properties. *Macromol. Rapid Commun.* 31 1163–1169. 10.1002/marc.200900876 21590870

[B198] Zheng Y. Li S. Weng Z. Gao C. (2015). Hyperbranched polymers: advances from synthesis to applications. *Chem. Soc. Rev.* 44 4091–4130. 10.1039/c4cs00528g 25902871

[B199] Zhou Q. Zhang L. Yang T. Wu H. (2018). Stimuli-responsive polymeric micelles for drug delivery and cancer therapy. *Int. J. Nanomed.* 13 2921–2942. 10.2147/IJN.S158696 29849457 PMC5965378

[B200] Zhu L. Perche F. Wang T. Torchilin V. P. (2014). Matrix metalloproteinase 2-sensitive multifunctional polymeric micelles for tumor-specific co-delivery of siRNA and hydrophobic drugs. *Biomaterials* 35 4213–4222. 10.1016/j.biomaterials.2014.01.060 24529391 PMC3981970

[B201] Zhu L. Wang T. Perche F. Taigind A. Torchilin V. P. (2013). Enhanced anticancer activity of nanopreparation containing an MMP2-sensitive PEG-drug conjugate and cell-penetrating moiety. *Proc. Natl. Acad. Sci. U.S.A.* 110 17047–17052. 10.1073/pnas.1304987110 24062440 PMC3801051

[B202] Zielinska A. Carreiro F. Oliveira A. M. Neves A. Pires B. Venkatesh D. N. (2020). Polymeric nanoparticles: production, characterization, toxicology and ecotoxicology. *Molecules* 25:3731. 10.3390/molecules25163731 32824172 PMC7464532

